# *Opsin1* regulates light-evoked avoidance behavior in *Aedes albopictus*

**DOI:** 10.1186/s12915-022-01308-0

**Published:** 2022-05-13

**Authors:** Xinyi Liu, Shuzhen Yang, Yuan Yao, Si Wu, Pa Wu, Zongzhao Zhai

**Affiliations:** Hunan Provincial Key Laboratory of Animal Intestinal Function and Regulation, Hunan International Joint Laboratory of Animal Intestinal Ecology and Health, College of Life Sciences, Hunan Normal University, Changsha, 410081 Hunan China

**Keywords:** *Opsin1*, *Aedes albopictus*, *Aedes aegypti*, *Culex quinquefasciatus*, Photobehavior, Vision, Light preference, Biting behavior, Blood feeding, Compound eyes

## Abstract

**Background:**

Mosquitoes locate a human host by integrating various sensory cues including odor, thermo, and vision. However, their innate light preference and its genetic basis that may predict the spatial distribution of mosquitoes, a prerequisite to encounter a potential host and initiate host-seeking behaviors, remains elusive.

**Results:**

Here, we first studied mosquito visual features and surprisingly uncovered that both diurnal (*Aedes aegypti* and *Aedes albopictus*) and nocturnal (*Culex quinquefasciatus*) mosquitoes significantly avoided stronger light when given choices. With consistent results from multiple assays, we found that such negative phototaxis maintained throughout development to adult stages. Notably, female mosquitoes significantly preferred to bite hosts in a shaded versus illuminated area. Furthermore, silencing *Opsin1*, a G protein-coupled receptor that is most enriched in compound eyes, abolished light-evoked avoidance behavior of *Aedes albopictus* and attenuated photonegative behavior in *Aedes aegypti*. Finally, we found that field-collected *Aedes albopictus* also prefers darker area in an *Opsin1*-dependent manner.

**Conclusions:**

This study reveals that mosquitoes consistently prefer darker environment and identifies the first example of a visual molecule that modulates mosquito photobehavior.

**Supplementary Information:**

The online version contains supplementary material available at 10.1186/s12915-022-01308-0.

## Background

Mosquitoes bite for animal blood to sustain their reproduction, but concomitantly transmit various human diseases including dengue fever, yellow fever, and malaria, thus posing global threat to humans and public health. They integrate various sensory systems to combine olfactory, thermoreceptive, and visual stimuli in order to detect, identify, and locate a specific host and acquire a bloodmeal [1–3]. Studies have elucidated how carbon dioxide, thermo, and host body odor activate host-seeking behavior of mosquitoes, with the corresponding chemical receptors and downstream signaling molecules identified [3–10]. However, visual features controlling mosquito light preference and target-seeking behavior have only begun to be addressed very recently [3, 11, 12].

Many insects display strong spectral sensitivity for short-wavelength light. Such light response mediated by insect compound eyes is critical to shape physiological adaption and specific behavioral features underlying insect survival and reproduction. The *Aedes aegypti* compound eyes is composed of ommatidia each containing eight photoreceptors (R1-8), with R1-7 forming a circular structure and R8 localized in the center [13]. Each photoreceptor cell possesses a microvillar rhabdomere, the place where photoreception and phototransduction take place [14]. While microvillar rhabdomeres of R1-6 and R8 cells are attached to neighboring rhabdomeres to create a fused rhabdom, presumably leading to enhanced light sensitivity, R7 cell has a small rhabdomere located at the distal surface of the fused rhabdom [13]. Embedded in rhabdomere membranes are Opsins, a class of seven transmembrane-containing GPCR proteins coupled to a light-sensitive chromophore that initiate phototransduction [15]. Although *Ae. aegypti* is diurnal and *Anopheles gambiae* is nocturnal, the two species share similar photoreceptor organization [13].

Bioinformatic analysis followed by manual annotation has identified thirteen Opsins in *Culex quinquefasciatus* and eleven Opsins in *An. gambiae* [16], while the genome of *Ae. aegypti* encodes ten potential *opsin* genes [17]. Different *opsin* genes show a unique expression pattern. In *Ae. aegypti* larvae, *Opsin3* is expressed in the majority of photoreceptors while *Opsin7* is expressed in two small clusters of photoreceptors located within the satellite and central stemmata [18]. In the compound eyes of adult *Ae. aegypti*, *Opsin1* is expressed in R1-6 and R8 cells, and interestingly, *Opsin2* and *Opsin8* are expressed exclusively in R7 cells that separate from the fused rhabdom [13, 19]. Additionally, *Opsin9* is expressed in all R7 cells and a subset of R8 cells located in the dorsal region of adult *Ae. aegypti* [14]. Although studies have revealed the expression pattern and light-triggered movement of Opsins in photoreceptors of both larval and adult mosquito retina [13, 14, 18–21], there is limited functional insight into the roles of mosquito *opsin* genes.

In this study, we characterized the photobehavior of *Ae. aegypti*, *Ae. albopictus*, and *Cx. quinquefasciatus* at different developmental stages and revealed a crucial role of *Opsin1* in controlling innate light preference of adult female *Ae. albopictus*.

## Results

### Adult female mosquitoes are photonegative

**Fig. 1:**
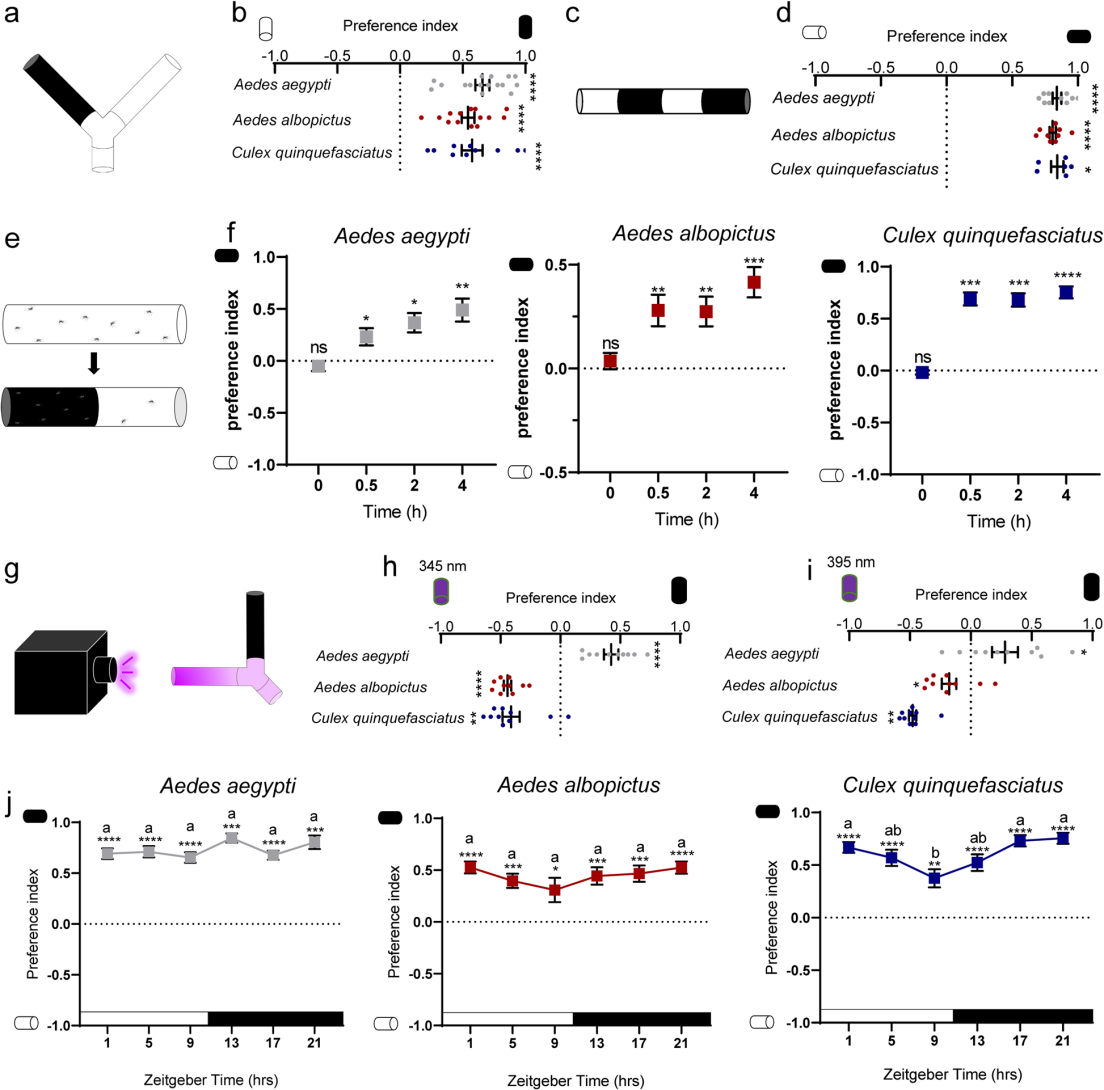
Photobehavior of adult female mosquitoes. **a**, **b** Y-maze assay. **a** Assay schematic. **b** Preference index between illuminated and shaded environment (*n* = 400, 350, 250 females). **c**, **d** Tube assay. **c** Assay schematic and **d** preference index between illuminated and shaded environment (*n* = 250, 250, 150 females). **e**, **f** Shading assay. **e** Assay schematic. **f** Photobehavior of *Ae. aegypti*, *Ae. albopictus*, and *Cx. quinquefasciatus* upon shading (*n* = 209, 325, 246 females). **g**–**i** Photobehavior assay with blacklight. **g** Assay schematic. **h** Photobehavior assay with UV light of 345 nm performed at ZT17-ZT23 (*n* = 250 females per species). **i** Photobehavior assay with UV light of 395 nm performed at ZT17-ZT23 (*n* = 250 females per species). **j** Photobehavior of adult female mosquitoes across zeitgeber time. Photobehavior of *Ae. aegypti*, *Ae. albopictus*, and *Cx. quinquefasciatus* across zeitgeber time (*n* = 450 females per species). Mosquitoes were maintained on 12 h light/12 h dark cycles, with dark periods highlighted in black. Kruskal-Wallis test, followed by Dunn’s multiple comparisons test was performed for testing among different groups for *Ae. aegypti*. One-way ANOVA, followed by Tukey’s multiple comparisons test was performed for testing among different groups for *Ae. albopictus* and *Cx. quinquefasciatus*. Data labelled with different lowercase letters are significantly different from each other. Experimental groups denoted by “ab” are not significantly different from either “a” or “b” groups. Data includes over three biological repeats, each with four technical replicates. **b**, **d**, **h**–**i** Dots represent PI of individual repeats, which includes over three biological repeats, each with two technical replicates. **b**, **d**, **f**, **h**–**j** Data are presented as mean ± SEM. Total number of mosquitoes released are indicated. Photobehavior was analyzed using one sample *t* test or Wilcoxon signed-rank test for tests against chance. *P < 0.05, **P < 0.01, ***P < 0.001, ****P < 0.0001, ns: not significant

While various behavioral paradigms and setting have provided information on *Drosophila* photobehavior [22], we adopted three different assays to characterize the preference of adult mosquito for illuminated versus shaded environment. In the Y-maze assay (Fig. 1a), mosquitoes were released to make choice between illuminated and shaded arms, and preference index (PI) was calculated as the number of mosquitoes that preferred the shaded chamber subtracting the number that preferred the illuminated chamber and further divided by the total number of mosquitoes that made a choice. In contrast to previous reports [23], *Ae. aegypti*, *Ae. albopictus*, and *Cx. quinquefasciatus* all exhibited strong preference for darker environment (Fig. 1b, Additional file 1: Video S1). These observations are actually more striking when considering the fact that diurnal (*Ae. aegypti* and *Ae. albopictus*) and nocturnal (*Cx. quinquefasciatus*) mosquitoes both showed light-evoked avoidance behavior (Fig. 1b). When host cues were presented in both arms of the Y-maze, female mosquitoes still preferred shaded over light-exposed side (Additional file 2: Fig. S1a-b). We further measured the light choice using a previously described tube assay [22] with modifications (Fig. 1c) and confirmed our conclusion that adult mosquitoes are photonegative (Fig. 1d, Additional file 3: Video S2). In the abovementioned assays, mosquitoes were released from mesh cages to make a quick choice (Additional file 2: Fig. S1c-d). To further support our conclusion, mosquitoes in resting state were tested for light preference. We carefully shaded half of the illuminated tube with mosquitoes rested in and monitored mosquito distribution for hours (Fig. 1e). Mosquitoes that initially distributed evenly in the tube slowly migrated to shaded environment within 4 h (Fig. 1f).

While previous studies reported behavioral attraction to ultraviolet (UV) light for *Aedes*, *Anopheles*, and *Culex* in semi-field setting and in field conditions at night [24, 25], we extended our Y-maze assay to study mosquito photobehavior to UV light versus darkness (Fig. 1g). *Cx. quinquefasciatus* and *Ae. albopictus* were found to exhibit consistent phototactic attraction to UV light of both 395 nm and 345 nm (Fig. 1h, i). However, *Ae. aegypti* exhibited phototactic avoidance to UV light of both 395 nm and 345 nm (Fig. 1h, i). Taken together, our Y-maze assay argues that the phototaxis of *Aedes*, *Anopheles*, and *Culex* to UV light is more complex than originally believed, in sharp contrast to a consistent and robust avoidance of visible light by all the three species.

Since mosquito behaviors are guided by circadian rhythms [26], we investigated the photobehavior of the *Aedes* and *Culex* mosquitoes across zeitgeber time (ZT). Mosquitoes were entrained under 12h:12h light:dark cycles and subsequently presented with a choice of illuminated versus shaded environments in a Y-maze assay at indicated ZT time points. Our data indicated that *Ae. aegypti*, *Ae. albopictus*, and *Cx. quinquefasciatus* maintained robust photonegative behavior across all ZTs (Fig. 1j). Collectively, our results show that both daytime-active and nighttime-active mosquitoes are innately photonegative across the length of a day.

**Fig. 2:**
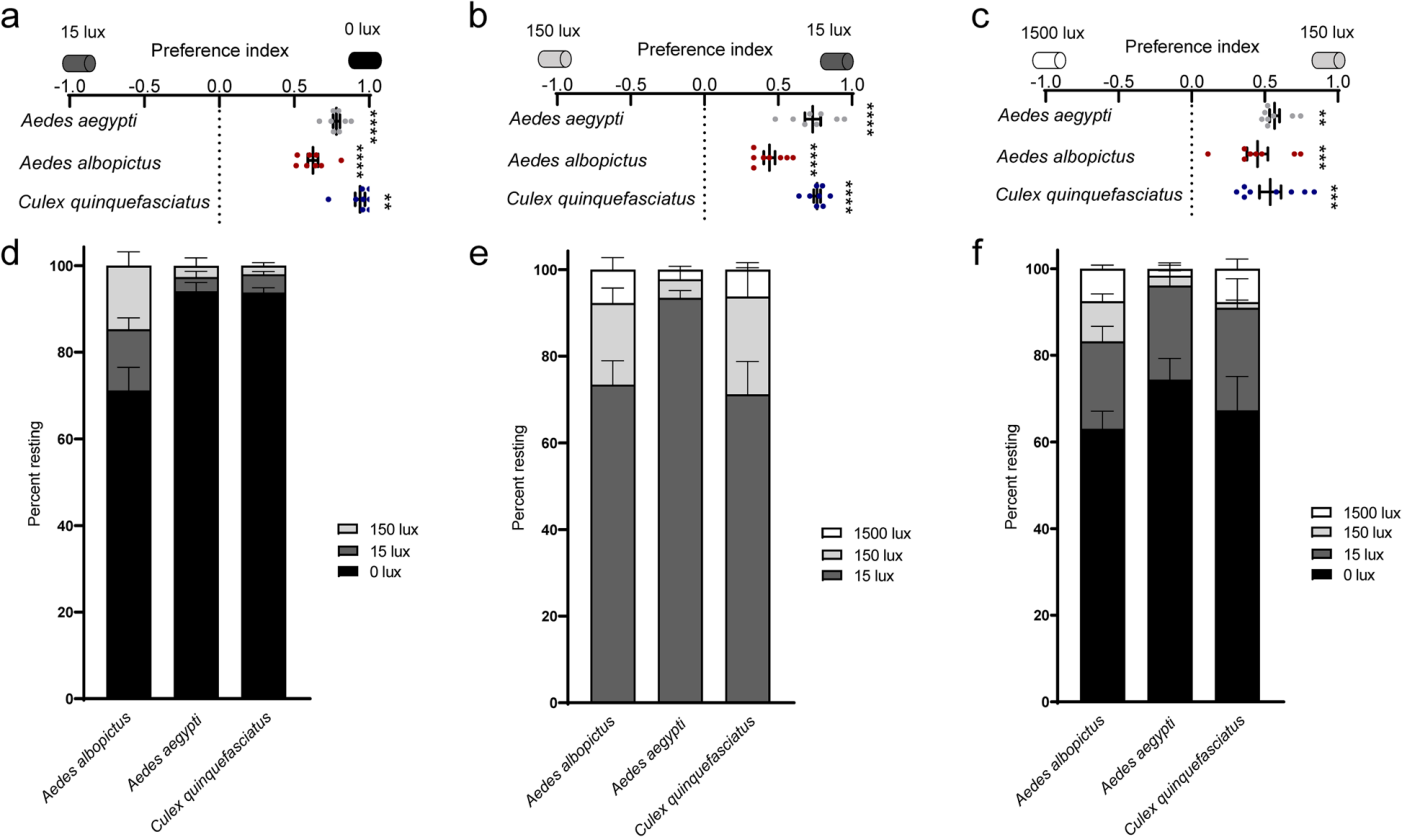
Mosquito preference index over different illumination intensities. **a**–**c** Binary photopreference. **a** Photopreference between 0 lux and 15 lux (*n* = 200 females per species). **b** Photopreference between 15 lux and 150 lux (*n* = 200 females per species) and **c** photopreference between 150 lux and 1500 lux (*n* = 200 females per species). **d**, **e** Trinary photopreference. **d** Photopreference over 0 lux, 15 lux and 150 lux (*n* = 150 females per species) and **e** photopreference over 15 lux, 150 lux, and 1500 lux (*n* = 150 females per species). **f** Quaternary photopreference. Histogram showing mosquito photopreference over 0 lux, 15 lux, 150 lux, and 1500 lux (*n* = 200 females per species). **a**–**c** Dots represent PI of individual repeats, which includes over three biological repeats, with two technical replicates for each biological repeat. Photobehavior were analyzed using one sample *t* test or Wilcoxon signed-rank test for tests against chance. **P < 0.01, ***P < 0.001, ****P < 0.0001. **a**–**f** Data are presented as mean ± SEM. Total number of mosquitoes used are indicated

We cannot so far rule out the possibility that mosquitoes prefer light of certain intensities. To better reveal their phototaxis features, we measured light preference with different levels of illumination intensity available ranging from 0 lux, 15 lux, 150 lux, and 1500 lux, which correspond to darkness, dim light, home lighting, and extremely bright conditions, respectively. In binary choice assays (Additional file 2: Fig. S2a-c), mosquitoes consistently preferred environment with illumination of lower intensities, choosing 0 lux over 15 lux, 15 lux over 150 lux, and 150 lux over 1500 lux (Fig. 2a–c). Trinary preference assay (Additional file 2: Fig. S2d-e) revealed that over 65% female mosquitoes of all the three species preferred the darkest environment available (Fig. 2d, e). In quaternary choice assay (Additional file 2: Fig. S2f), over 60% adult female mosquitoes preferred complete darkness and around 20% mosquitoes preferred dim light (Fig. 2f). Our data collectively suggests that adult female mosquitoes exhibited light-evoked avoidance behavior and consistently exploited darker environment.

### Mosquitoes are photonegative throughout all developmental stages

**Fig. 3:**
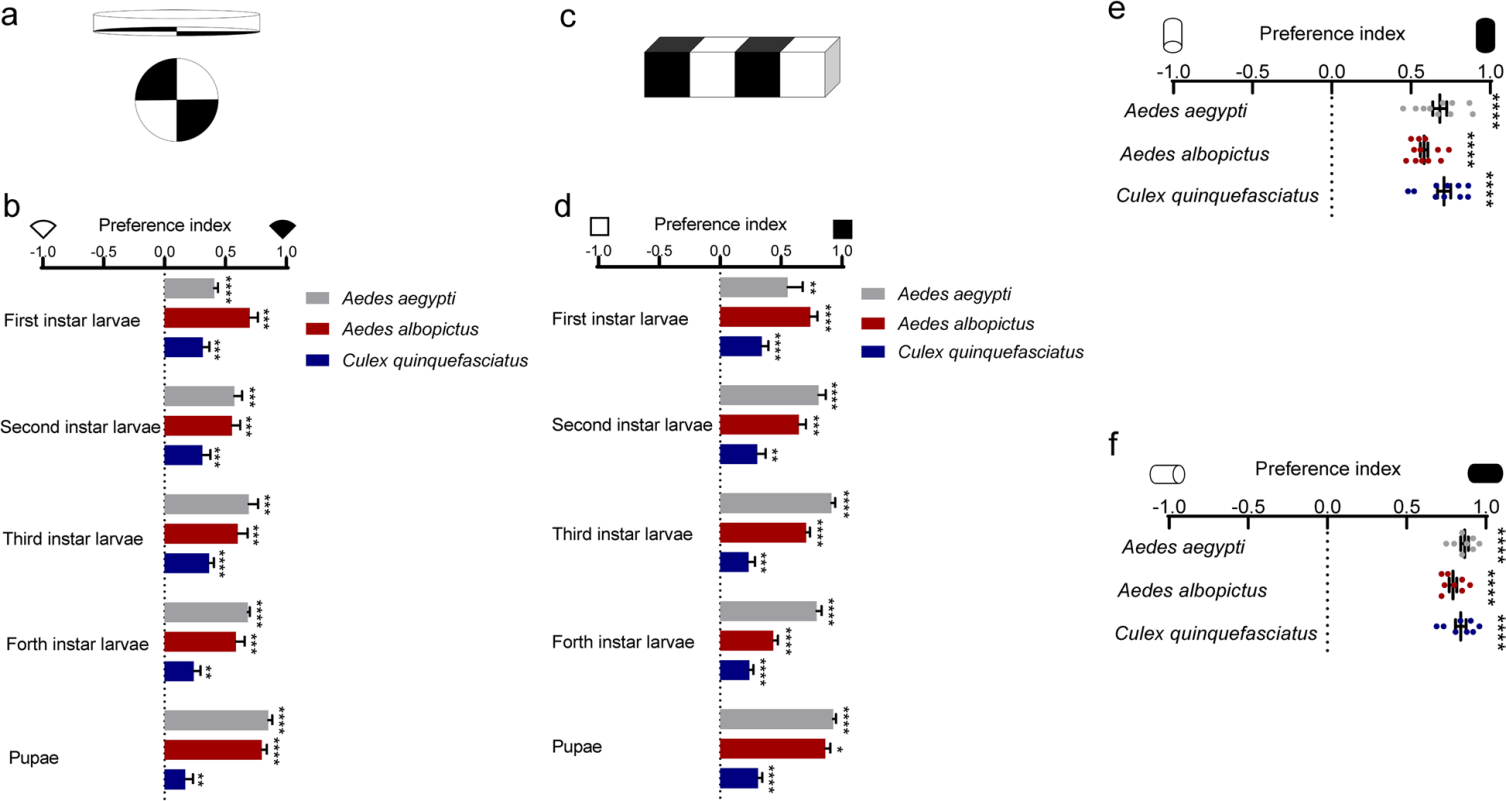
Photobehavior of larvae, pupae and adult male mosquitoes. **a**–**d** Plate assay (**a**, **b**) and tray assay (**c**, **d**) to test photobehavior of larvae and pupae. **a**, **c** Assay schematic. Fifty larvae or pupae were released in the center of the testing plate/tray in each individual test. **b**, **d** Preference index of *Ae. aegypti*, *Ae. albopictus*, and *Cx. quinquefasciatus* between illuminated and shaded environment (*n* = 150 larvae or pupae per group for *Ae. aegypti* and *Ae. albopictus*, *n* = 300 larvae or pupae per group for *Cx. quinquefasciatus*). **e**, **f** Y-maze assay (*n* = 250, 300, 250 males) (**e**) and tube assay (*n* = 200 males per species) (**f**) to test photobehavior of male mosquitoes. **b**–**d** Data are presented as mean ± SEM. Total number of larvae or pupae tested are indicated. **e**, **f** Dots represent PI of individual repeats, which includes over three biological repeats, with two technical replicates for each biological repeat. Total number of mosquitoes tested are indicated. **b**–**f** Photobehavior were analyzed using one sample *t* test or Wilcoxon signed-rank test. *P < 0.05, **P < 0.01, ***P < 0.001, ****P < 0.0001

The fruitfly *Drosophila melanogaster* changes light preference during development [22, 27, 28]. We also investigated mosquito innate response to light during larval and pupal stages using a previously described plate assay [29] with modifications (Fig. 3a). Similar to adults, larvae and pupae migrated towards the darker quadrants (Fig. 3b, Additional file 4: Video S3, Additional file 5: Video S4). Additionally, the negative phototaxis of larvae and pupae was confirmed with a tray assay (Fig. 3c, d, Additional file 6: Video S5, Additional file 7: Video S6). In these assays, larvae and pupae showed swarming behavior and tended to gather stochastically in one of the two dark areas in each independent test; however, the overall distribution among the two dark quadrants was similar in the plate assay (Additional file 2: Fig. S3a-c). Interestingly, in the tray assay, larvae and pupae significantly preferred dark region and larvae significantly swarmed at the edge (Additional file 2: Fig. S3d-f).

As females were photonegative at adult stage (Fig. 1b, d, and f), we further studied the photobehavior of males with the abovementioned assays. Male mosquitoes were found to exhibit significant aversion to light in both Y-maze assay (Fig. 3e) and tube assay (Fig. 3f). Therefore, mosquitoes are photonegative independent of their stages and sexes. Hereafter, we characterized the light-evoked avoidance behaviors only in female adults, which transmit human pathogens via bloodmeal and possess threat to public health.

### Biting behaviors of adult mosquitoes are instructed by light

**Fig. 4:**
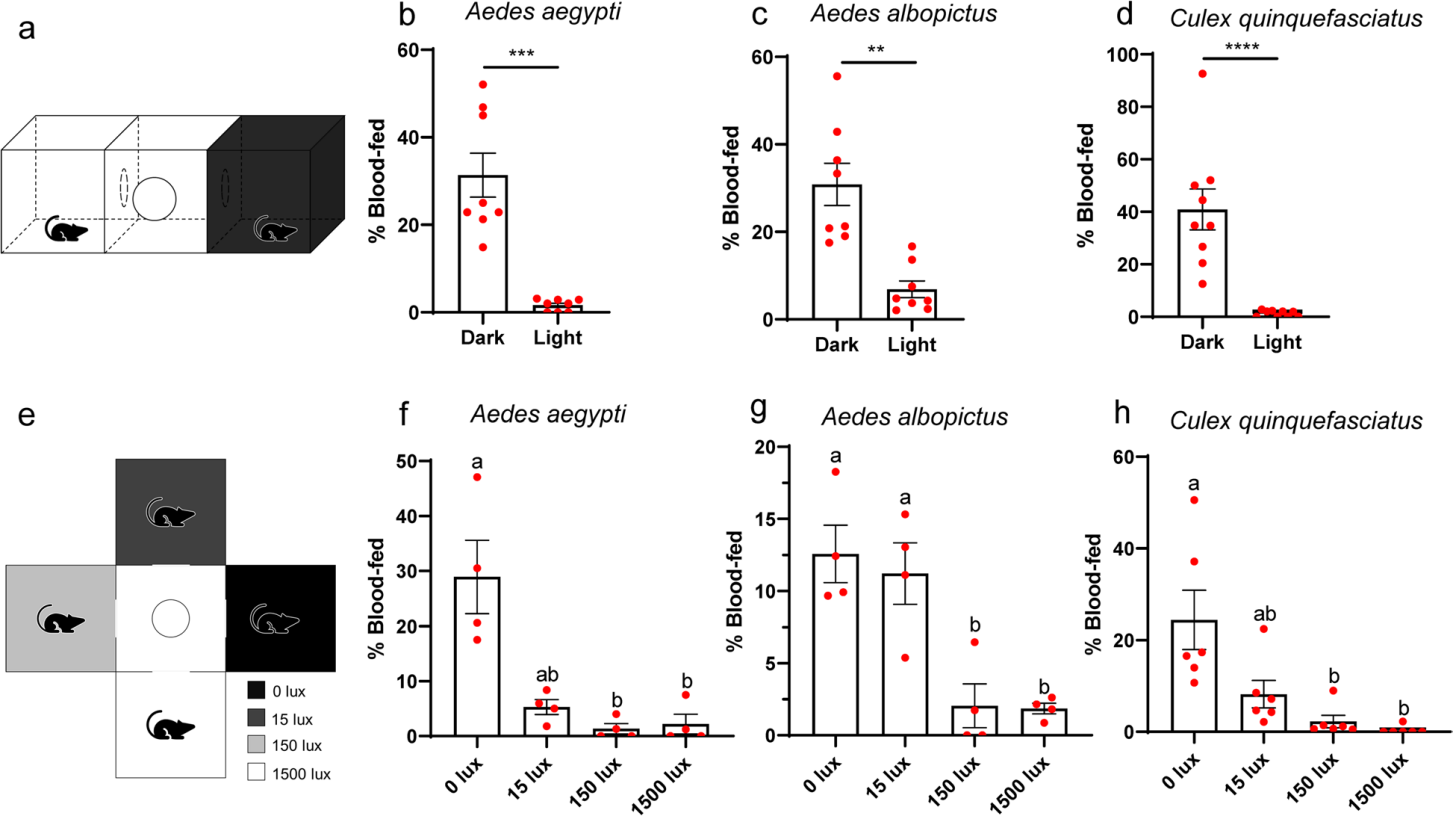
Biting behavior of female mosquitoes under different light intensities. **a**–**d** Biting preference between hosts from illuminated and shaded environment. **a** Assay schematic and **b**–**d** percent of *Ae. aegypti* (**b**), *Ae. albopictus* (**c**), and *Cx. quinquefasciatus* (**d**) that bitted hosts in illuminated and shaded environment (*n* = 400, 400, 450 females). **e**–**h** Biting preference for hosts in environment with different illumination intensities. **e** Assay schematic and **f**–**h** percent of *Ae. aegypti* (**f**), *Ae. albopictus* (**g**), and *Cx. quinquefasciatus* (**h**) that bitted hosts in environment with indicated illumination intensities (*n* = 800, 800, 1200 females). **b**–**d**, **f**–**h** Data are presented as mean ± SEM. Each dot represents the blood feeding rate of each biological repeat. Total number of mosquitoes used are indicated. The data are presented as the number of fully engorged mosquitoes that bitted host in environment with indicated illumination intensities in relative to the total mosquitoes released. **b**–**d** Mann-Whitney test (**b**, **d**) or unpaired *t* test with Welch’s correction (**c**) was performed for testing between two groups. **P < 0.01, ***P < 0.001, ****P < 0.0001. **f**, **h** Kruskal-Wallis test, followed by Dunn’s multiple comparisons test was performed for testing among different groups. **g** One-way ANOVA, followed by Tukey’s multiple comparisons test was performed for testing among different groups. **f**–**h** Different lowercase letters indicate significantly different. Experimental groups denoted by “ab” are not significantly different from either “a” or “b” groups

To investigate whether light perception modulates biting behaviors of mosquitoes, we adopted a biting assay in which female mosquitoes are exposed directly to live hosts (Fig. 4a). Significantly higher ratio of *Ae. aegypti* (Fig. 4b), *Ae. albopictus* (Fig. 4c), and *Cx. quinquefasciatus* (Fig. 4d) preferred biting mice in dark over illuminated environment. Since mosquitoes in the field likely make choices between multiple illumination intensities, we further applied a multi-chamber biting assay. Mosquitoes were allowed to navigate between four chambers of different illumination intensities each housing a host (Fig. 4e-h). In this assay, significantly higher ratio of *Ae. aegypti* and *Cx. quinquefasciatus* preferred blood feeding in complete darkness as compared with illumination intensities of both 150 lux and 1500 lux (Fig. 4f, h). In contrast, significantly higher ratio of *Ae. albopictus* preferred both 0 lux (darkness) and 15 lux (dim light) as compared with the other two illumination intensities (Fig. 4g). Interestingly, blood feeding ratio was not significantly different among mosquitoes under different illumination intensity under condition that mosquitoes were not given alternative choices (Additional file 2: Fig. S4). Collectively, these results support that mosquitoes preferred hosts in dark environment.

### Photobehavior of *Aedes albopictus* was associated with molecules in the compound eyes

**Fig. 5:**
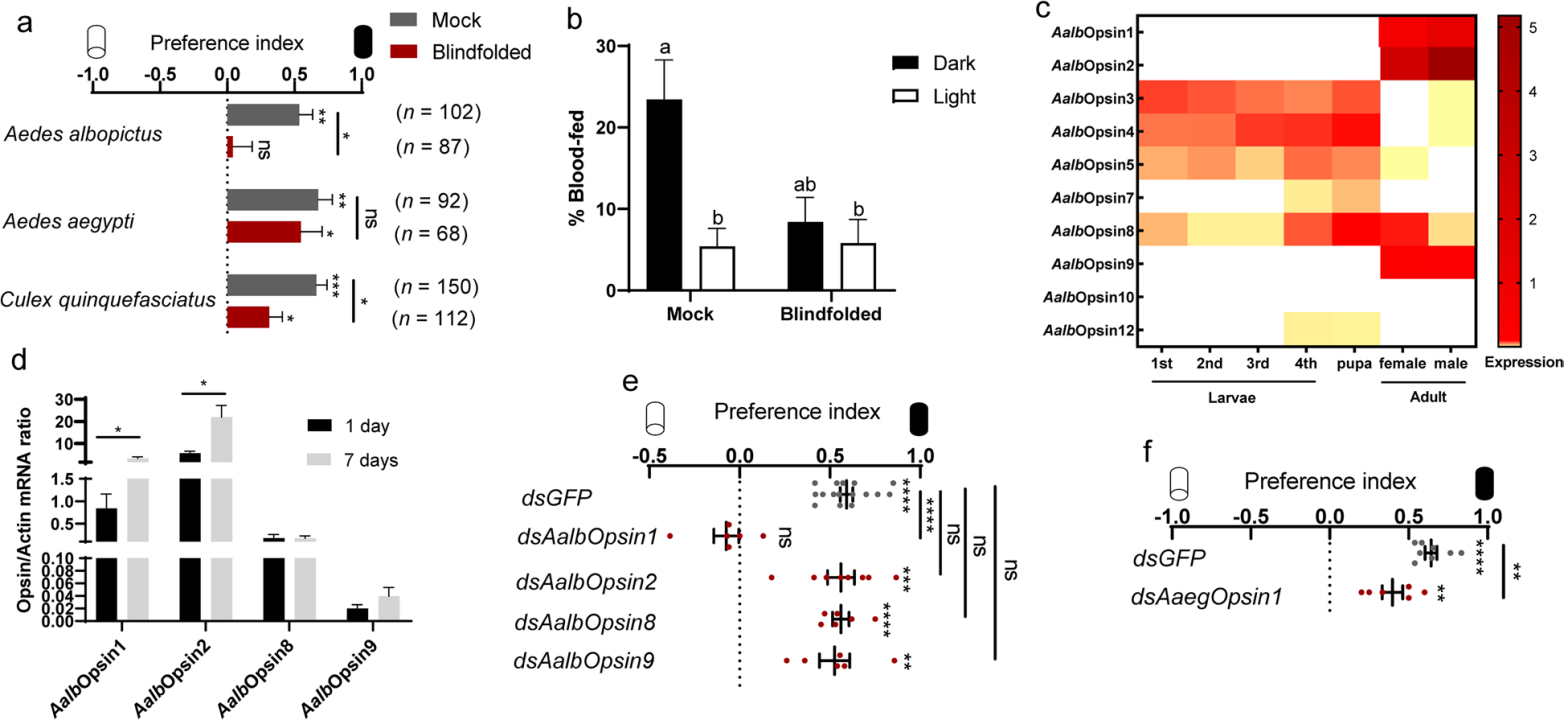
Role of Opsins in mosquito photobehavior. **a** Effect of eye occlusion on mosquito photobehavior. **b** Effect of eye occlusion on *Ae. albopictus* biting preference (Mock, *n* = 104 females; Blindfolded, *n* = 65 females). Data is presented as the number of fully engorged mosquitoes that bitted host in illuminated or shaded environment relative to the total number of mosquitoes released. Two-way ANOVA, followed by Tukey’s multiple comparisons test was performed for testing among different groups. Different lowercase letters are significantly different. Experimental groups denoted by “ab” are not significantly different from either “a” or “b” groups. **c** Heatmap showing expression of *opsin* genes in *Ae. albopictus* across developmental stages. Expression levels of *opsin* genes in mixed-gender first instar larvae (1st), mixed-gender second instar larvae (2nd), mixed-gender third instar larvae (3rd), mixed-gender forth instar larvae (4th), mixed-gender pupae, adult females, and adult males. *n* = 50 for first and second instar larvae; *n* = 30 for third instar larvae; *n* = 20 for forth instar larvae and pupae; *n* = 3 for adult female and male. **d** *Opsin* gene expression of adult females at 1 day and 7 days after eclosion. Expression levels of *opsin* genes were normalized against *Ae*. *albopictus actin* (*AALF010408*). *n* = 9 females per group. **e**, **f** Effects of knocking down *opsin* genes on mosquito photobehavior. **e** Preference index for shaded area upon knocking down indicated *opsin* genes in *Ae. albopictus* (ds*GFP*, *n* = 328 females; ds*AalbOpsin1*, *n* = 103 females; ds*AalbOpsin2*, *n* = 166 females; ds*AalbOpsin8*, *n* = 88 females; ds*AalbOpsin9*, *n* = 95 females). **f** Effect of knocking down *Opsin1* on *Ae. aegypti* photobehavior (ds*GFP*, *n* = 126 females; ds*AaegOpsin1*, *n* = 72 females). **a**–**f** Data are presented as mean ± SEM. Total number of female mosquitoes tested for each group was indicated by *n* in the figure. Photobehavior was analyzed using one sample *t* test. **a**, **d**, **f** Unpaired *t* test was performed for testing among different groups. **e** One-way ANOVA, followed by Tukey’s multiple comparisons test was performed for testing among different groups. *P < 0.05, **P < 0.01, ***P < 0.001, ns: not significant

To uncover the mechanisms that underlie the photonegative behavior, we first blindfolded mosquitoes to test if the compound eyes are mediating the photobehavior, using methods previously described for *Drosophila melanogaster* [30–32]. As expected, ocularly painted *Ae. albopictus* were unable to differentiate between illuminated and shaded area, showing that eye occlusion completely abolished the photonegative behavior of *Ae. albopictus* (Fig. 5a). Surprisingly, painting the eyes, which attenuated approximately 99.14% light flux tested using glass coverslips painted in the same way, failed to abolish the photonegative behavior of female *Ae. aegypti* and *Cx. quinquefasciatus* (Fig. 5a). We further investigated the biting behavior of blindfolded *Ae. albopictus* with a binary choice of host in illuminated versus shaded chamber (Fig. 4a). While *Ae. albopictus* with normal vision preferred hosts in dark environment, similar amount of ocularly occluded *Ae. albopictus* bit hosts from a dark and illuminated environment (Fig. 5b), indicating that biting behavior of *Ae. albopictus* was modulated by light flux via compound eyes.

### Knocking down *AalbOpsin1* abolished negative phototaxis of *Aedes albopictus*

Opsin, the light receptor in photoreceptors of the retina, plays a decisive role in visual perception [33]. Genome sequencing and bioinformatic analysis revealed ten *opsin* genes in *Ae. aegypti* and thirteen *opsin* genes in *Cx. quinquefasciatus* [16, 17]. Ten *opsin* genes were manually annotated in the genome of *Ae. albopictus* via homologous comparison with those of *Ae. aegypti* and denoted as *AalbOpsin1-10* (Additional file 8: Table S1). Phylogenetic analyses were performed with Opsins from *Ae. albopictus*, *Ae. aegypti*, *Cx. quinquefasciatus*, and *D. melanogaster* to discern their evolutionary relationship (Additional file 2: Fig. S5). The results suggest that Opsins of *Ae. albopictus* and *Ae. aegypti* are phylogenetically close, while Opsins are quite divergent among different genera. Furthermore, we characterized the expression of all the rhabdomeric *opsin* genes present in *Ae. albopictus*. While *AalbOpsin3*, *AalbOpsin4*, and *AalbOpsin5* were highly expressed in larvae, their expression decreased along with development (Fig. 5c). By contrast, *AalbOpsin1*, *AalbOpsin2*, *AalbOpsin8*, and *AalbOpsin9* had a relatively low level of expression at the larval and pupal stage, but were highly expressed in adults of *Ae. albopictus* (Fig. 5c). We further characterized the expression of Opsins that specifically enriched in adults of *Ae. albopictus* and found that the Opsins were abundant at 1 day post emergence and the expression of *AalbOpsin1* and *AalbOpsin2* were further upregulated at 7 days post emergence (Fig. 5d).

To reveal the functional aspects of Opsins, we initially performed a screen of Opsins with high expression level in females of *Ae. albopictus* by silencing each gene with double-stranded RNA (dsRNA) via thoracic injection. Knockdown of *AalbOpsin1* with ds*AalbOpsin1*^*#1*^ completely abolished the photonegative behavior of *Ae. albopictus*, while silencing *GFP* (as negative control), *AalbOpsin2*, *AalbOpsin8*, and *AalbOpsin9* had no discernable effect (Fig. 5e, Additional file 2: Fig. S6a-j). Of note, knocking down *Aalb*Opsin2, which shares 87.94% similarity with *Aalb*Opsin1 (Additional file 2: Fig. S6k), was unable to change the photophobic behavior of *Ae. albopictus*. We then checked if *AaegOpsin1*, which shares 93.85% similarity with *AalbOpsin1*(Additional file 2: Fig. S6l), plays a role in the photobehavior of *Ae. aegypti.* Silencing of *AaegOpsin1* led to a decrease of the photonegative behavior of *Ae. aegypti*, although an overall but weak preference for dark regions was still maintained (Fig. 5f, Additional file 2: Fig. S6m).

**Fig. 6:**
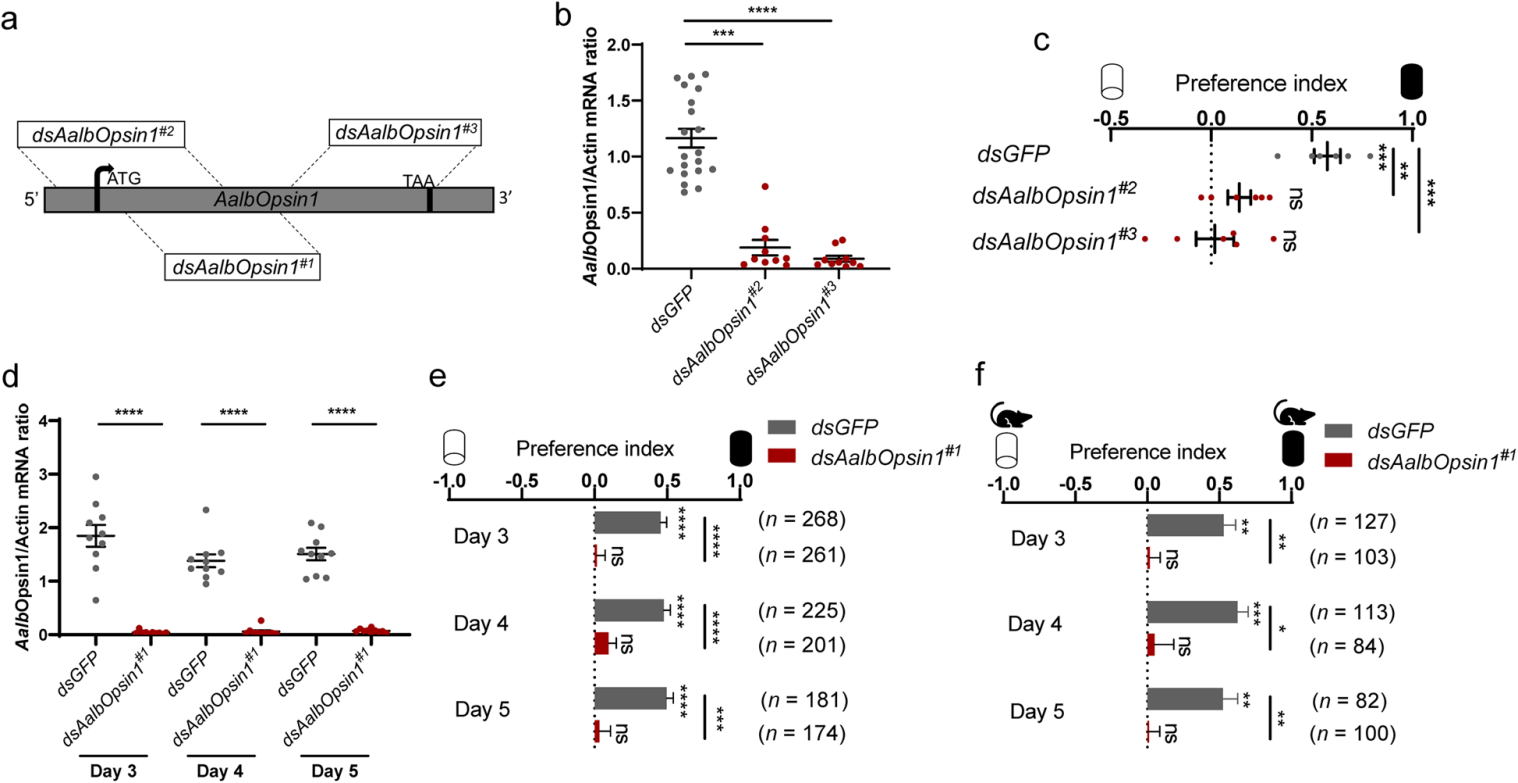
Photobehavior of *opsin1*-silenced *Ae. albopictus*. **a**–**c** Photobehavior of *Ae. albopictus* injected with two additional dsRNA against *opsin1*. **a** Schematic depiction of the *Opsin1 *and the design of all the three dsRNAs used in this study (ds*AalbOpsin1*^*#1*^, ds*AalbOpsin1*^*#2*^, and ds*AalbOpsin1*^*#3*^). **b** Knockdown efficiency of ds*AalbOpsin1*^*#2*^ and ds*AalbOpsin1*^*#3*^ assayed with qPCR (each dot denotes one mosquito; ds*GFP*, *n* = 20 females; ds*AalbOpsin1*^*#2*^, *n* = 10 females; ds*AalbOpsin1*^*#3*^, *n* = 10 females). **c** Photobehavior of *Ae. albopictus* injected with ds*AalbOpsin1*^*#2*^ and *AalbOpsin1*^*#3*^ (ds*GFP*, *n* = 105 females; ds*AalbOpsin1*^*#2*^, *n* = 111 females; ds*AalbOpsin1*^*#3*^, *n* = 106 females). Dots represent PI of individual repeats, which includes three biological repeats, with two technical replicates for each biological repeat. **d**–**f** Photobehavior of *Ae. albopictus* injected with ds*AalbOpsin1*^*#1*^. **d ***AalbOpsin1* expression at 3–5 days after thoracic inoculation of ds*AalbOpsin1*^*#1*^ (each dot denotes one mosquito; *n* = 10 females per group). **e** Photobehavior of *Opsin1*-silenced *Ae. albopictus* with Y-maze assay. **f** Photobehavior of *opsin1*-silenced *Ae. albopictus* in the presence of host cues with Y-maze assay. **b**–**f** Data are presented as mean ± SEM. *n* in the figure denotes the total number of female mosquitoes tested for each group. Photobehavior was analyzed using one sample *t* test or Wilcoxon signed-rank test. **b**, **d** Expression levels of *opsin* were normalized against *Ae. albopictus actin* (*AALF010408*). **b** Kruskal-Wallis test, followed by Dunn’s multiple comparisons test was performed for testing among different groups. **c** One-way ANOVA, followed by Tukey’s multiple comparisons test was performed for testing among different groups. **d**–**f** Two-way ANOVA, followed by Sidak’s multiple comparisons test was performed for testing among different groups. *P < 0.05, **P < 0.01, ***P< 0.001, ****P < 0.0001, ns: not significant

To further validate the role of *AalbOpsin1* in photonegative behavior of *Ae. albopictus*, two additional dsRNA designated as ds*AalbOpsin1*^*#2*^ and ds*AalbOpsin1*^*#3*^ were synthesized (Fig. 6a). Injection of both dsRNA significantly downregulated *AalbOpsin1* expression (Fig. 6b) and further abolished photophobic behavior of *Ae. albopictus* (Fig. 6c). To check the duration of RNAi effect, *Ae. albopictus* injected with *AalbOpsin1* dsRNA were subjected to Y-maze assay consecutively for 3 days. While mosquitoes injected with ds*GFP* remained photophobic from third to fifth day post dsRNA delivery, the mosquitoes injected with ds*AalbOpsin1* became photoneutral, both in the present and absence of host cues (Fig. 6d-f). The finding that knocking down *AalbOpsin1* failed to abolish the preference of *Ae. albopictus* to bite host in shaded environment (Additional file 2: Fig. S6n), suggests additional molecules might be needed for preferring to bite hosts in shaded environment. Collectively, these results showed that *AalbOpsin1* knockdown alone is sufficient to abolish the photonegative behavior of *Ae. albopictus*.

### *Aalb*Opsin1 mediates light-evoked avoidance behaviors of field-collected *Ae. albopictus*

**Fig. 7:**
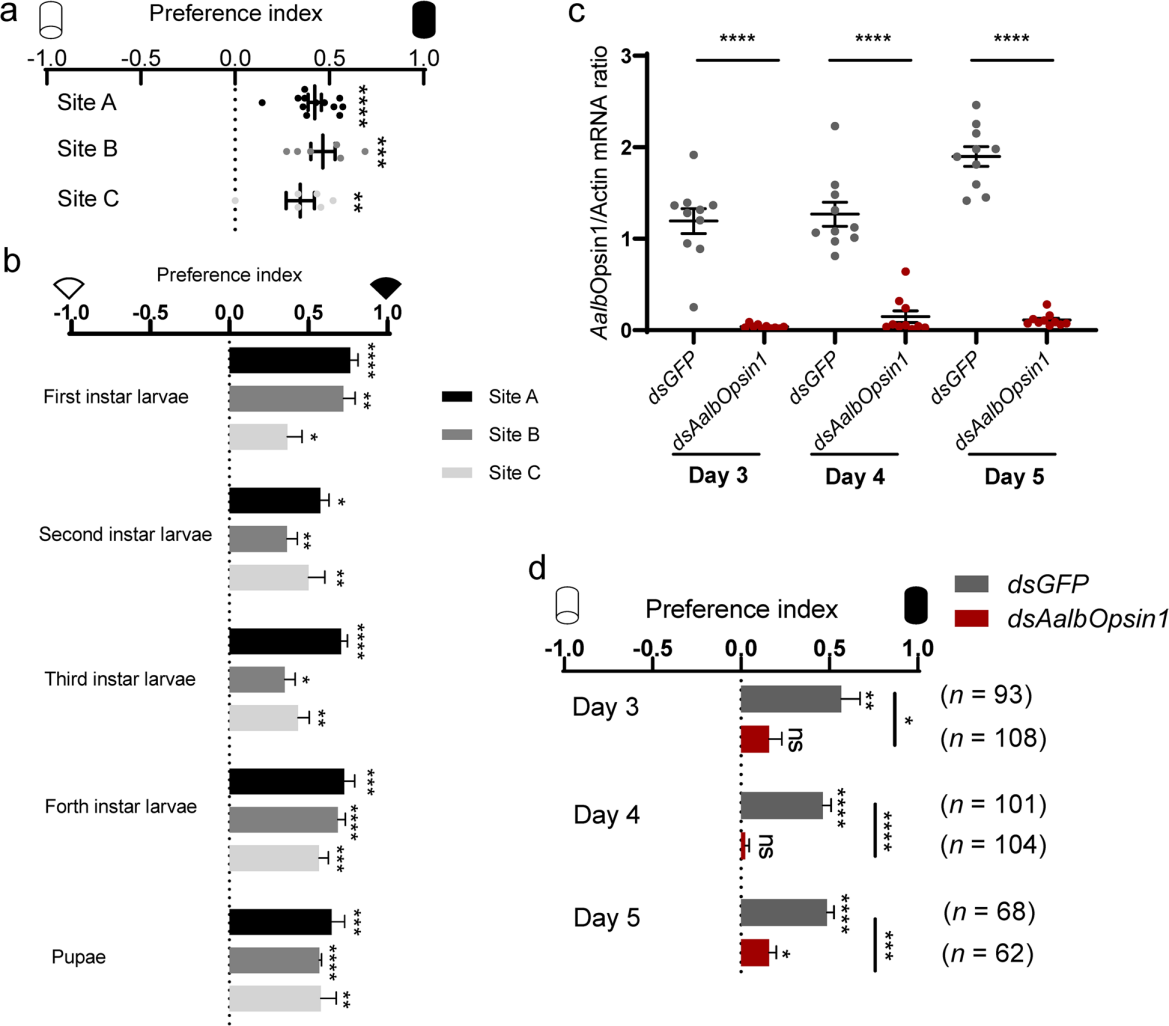
*Aalb*Opsin1 mediates negative phototaxis behavior of field-collected *Ae. albopictus. ***a**, **b** Photobehavior of field-collected *Ae. albopictus*. **a** Photobehavior of female adult (*n* = 300 females for site A, *n* = 150 females for site B and site C). **b** Photobehavior of field-collected larvae and pupae (*n* = 150 larvae or pupae per group for site A and site C, *n* = 100 first and third instar larvae for site B, *n* = 150 pupae for site B). **c**, **d** Photobehavior of field-collected *Ae. albopictus* injected with dsRNA against *AalbOpsin1*. **c** Knockdown efficiency of ds*AalbOpsin1* in field mosquitoes was measured with qPCR (each dot denotes one mosquito; *n* = 10 females per group). **d** Photobehavior of field-collected *Ae. albopictus* with *AalbOpsin1*-silenced. Starting from 3 days post-gene silencing, mosquito photobehavior was assessed with Y-maze assay. *n* in the figure denotes the total number of female mosquitoes tested for each group. **a**–**d** Data are presented as mean ± SEM. *n* denotes the total number of mosquitoes tested. Photobehavior was analyzed using one sample *t* test or Wilcoxon signed-rank test. **c**, **d** Two-way ANOVA, followed by Sidak’s multiple comparisons test was performed for testing among different groups. *p < 0.05, **p < 0.01, ***p < 0.001, ns: not significant

The aforementioned results indicate that *Aalb*Opsin1 is a critical protein in the compound eyes of *Ae. albopictus* for photophobic behavior. As mosquito physiology varies dramatically among strains and species [2, 34, 35], we next assessed the photobehavior of field-caught *Ae. albopictus*. Larvae, pupae, and adults of *Ae. albopictus* were collected from three different districts, including indoor and outdoor sites in villages, cities, and nearby natural environments (Additional file 2: Fig. S7a-b). Consistent with results obtained from laboratory strains, female adults of *Ae. albopictus* caught in different districts all exhibited photophobic behavior (Fig. 7a). Field-collected larvae and pupae also preferred shaded over illuminated environment (Fig. 7b, Additional file 2: Fig. S7c). To validate the role of *Aalb*Opsin1 in photophobic behavior of *Ae. albopictus*, we silenced *AalbOpsin1* in field-collected mosquitoes using RNAi. Adult *Ae. albopictus* emerged from field-collected larvae and pupae were injected with dsRNA and subjected to photobehavior assay for a consecutive measurement of 3 days. From third to fifth day post dsRNA delivery, silencing *AalbOpsin1* was always found to accompany with a consistent decrease of photonegative behavior in field-collected *Ae.* *albopictus* (Fig. 7c, d). Collectively, these results indicated a crucial role of *Aalb*Opsin1 in the innate light preference of field-caught *Ae. albopictus*.

## Discussion

Insect physiology and behaviors are guided by the sunlight. However, mechanistic insights into insect innate light preference are still lacking. With multiple experimental apparatuses, we found that *Ae. aegypti*, *Ae. albopictus*, and *Cx. quinquefasciatus* mosquitoes consistently avoid visible light and prefer to take a bloodmeal from hosts in the shaded environment.

Insect innate photobehavior can be exploited for vector control. While incandescent light bulb is being used to catch *Anopheles* in outdoor mosquito surveillance [36], laboratory experiments have shown that *An. gambiae* significantly preferred resting in darkness at both dusk and dawn [37]. *An. stephensi*, however, responds differently to light depending on sex and crepuscular period [37]. A recent study reported behavioral attraction to UV light for daytime-biting *Ae. aegypti* but behavioral avoidance of UV light by the nighttime-biting *Anopheles coluzzii* [23], contrasted to the finding in the field surveillance study whereby UV light attracted both *Anopheles* and *Culex* mosquitoes [24]. The discrepancy of behavioral response to visible and UV light may result from different source of light, circadian timing, and combined effect of sensory perception by mosquitoes in various studies. Our study critically conducted in the laboratory under well-controlled conditions has thus provided comprehensive data to shape our understanding of mosquito innate light preference. While our study shows that adult mosquitoes are generally photonegative, we also extended the photobehavior study to *Aedes* and *Culex* larvae and pupae using both plate assay and tray assay, and uncovered the same photophobic behavior, in line with a previous study that only recorded the light avoidance behavior of *Ae. aegypti* larvae [38]. We also noticed that larvae and pupae tend to swarm, which has been previously noticed and interpreted as gathering near preferred cues through random aggregation [39]. Interestingly, larvae tended to swarm at the edge of shaded area, an interesting phenomenon and warrants further study.

Previous studies have characterized the role of heat, odor, and CO_2_ levels in host-seeking and biting behavior of various mosquito species [8, 40]. However, it was not clear if light perception also influences biting behavior. Notably, our study showed that *Ae. aegypti*, *Ae. albopictus*, and *Cx. quinquefasciatus* consistently preferred to bite host in a dark area over host in an illuminated area, representing the first evidence of light-instructed host preference and biting behavior. To investigate whether the light preference and biting behavior are interrelated, we performed blood feeding assay under indicated illumination intensities in which mosquitoes were not given alternative choices for host. Unexpectedly, mosquito blood feeding rate was not significantly different under a gradient of illumination intensities. Additionally, painting the eyes of *Ae. albopictus*, which is known to abolish the photonegative behavior, led to equivalent blood feeding in dark and illuminated environment. Along the same line, knocking down *AalbOpsin1* to render *Ae. albopictus* photoneutral, did not affect preference to bite host in the dark region. Thus, the preference to bite in the darker environment when mosquitos are provided with choices of different light intensities is serendipitously driven by the photonegative behavior.

An intriguing question raised by our study is the different sensitivity of the compound eye photoreceptors in closely related mosquito species. Firstly, the same blindfolding assay was only able to completely abolish the photonegative behavior of *Ae. albopictus* but not that of *Ae. aegypti* and *Cx. quinquefasciatus*. The different outcomes of this blindfolding manipulation suggest that the compound eyes of *Ae. aegypti* and *Cx. quinquefasciatus* are highly sensitive and can still respond to the remaining light flux. Secondly, silencing of *Opsin1*, one of the major light receptors expressed in the adult mosquito eyes, completely abolished the light avoidance behavior of *Ae. albopictus*, while only lead to weak preference for dark regions of *Ae. aegypti*. The result, together with previous blindfolding results, suggest that *Ae. aegypti* might be highly sensitive to the light flux.

*Opsin1* and *Opsin2*, which are the most abundant Opsins in the *Aedes* with over 80% similarity, were recently found to play a redundant role in CO_2_-induced, vision-guided target attraction to black objects in *Ae. aegypti* [41]. Interestingly, our data showed that knocking down *Opsin1* rather than *Opsin2* abolished photonegative behavior of *Ae. albopictus*. Previous study revealed that *Opsin1* is expressed in all R1-6 photoreceptors and most R8 cells of the adult eye, while *Opsin2* is only expressed in R7 cells [13, 19, 21]. The different expression pattern may partially explain the discrepant requirement for *Opsin1* and *Opsin2* in the two *Aedes* species, suggesting functional divergence in different aspect of mosquito vision. Abolishing Opsin expression via CRISPR would be of importance to validate the results, especially for *Opsin2*, because RNAi was unable to completely abolish gene expression.

Photophobic behavior, which is regarded as an innate behavior that drives animals to choose an environment to maximize their survival fitness, has been widely observed in species of rodents and insects [28, 42, 43]. Sighted mice innately avoid illuminated areas, while *rd1* mice with photoreceptor degeneration lost the light avoidance behavior [44]. Photonegative behavior of *D. melanogaster* serves to keep larvae close to food source, to avoid desiccation and predation [29], and to further safeguard themselves by pupariating in the dark [28]. Thus, it is possible that light-evoked avoidance behavior allows mosquitoes to avoid desiccation under sunlight and reduce potential attack from host during biting, contributing to their adaption and survival. Remarkably, the three mosquito species tested showed consistent photophobic behavior throughout all developmental stages; in contrast, *D. melanogaster* reverses photobehavior during development [22, 27–29]. Further understanding of the genetic basis and the neurocircuit underlying the mosquito innate photonegative behavior should help design novel strategy of vector control and arbovirus disease prevention.

## Conclusions

In this work, we have quantified the response to light by both laboratory-reared and field-collected mosquitoes with multiple experimental settings (Y-maze assay, tube assay, Y-maze assay with host scent, and biting assay with two or multiple chambers), and found that mosquitoes consistently preferred a darker environment. Interestingly, *Ae. aegypti* and *Ae. albopictus*, which are diurnal, and *Cx. quinquefasciatus*, which is nocturnal, showed consistent photophobic behavior throughout the circadian cycle. For all the three species tested, adult females which transmit arbovirus via blood feeding significantly preferred host in a darker environment, which underlies a notable trait in both vector control and arbovirus prevention. In addition to adult females, negative phototaxis was validated in adult males and throughout developmental stages. Mechanistically, light receptor Opsin1 expressed in the insect compound eyes is essential for the photonegative behavior of *Ae. albopictus*. Collectively, this study presented novel insights into mosquito innate light preference and biting behavior and identifies the first example of a visual molecule that modulates mosquito innate photobehavior.

## Methods

### Mosquito rearing

*Ae. aegypti*, *Ae. albopictus*, and *Cx. quinquefasciatus* were obtained from Guangzhou Wolbaki Biotechnology Co., Ltd. Mosquito larvae were reared in dechlorinated tap water in plastic containers and raised with standard diet, which consisted of a 0.1% solution of three parts liver broth (CM0077, Oxoid) and two parts yeast extract (LP0021, Oxoid). Adult mosquitoes were maintained in a cage with unlimited access to water and sugar (raisin for standard diet). Mosquitoes were maintained in incubators (HWS-1000, Ningbojiangnan) with 12h:12h light:dark cycle at 27 °C with 75% humidity. Female mosquitoes aged 6–10 days post-eclosion were subjected for further investigation unless otherwise mentioned.

### Collection of field mosquitoes

We collected larvae, pupae, and adults from three different districts (Additional file 2: Fig. S7a) from June to September, 2021. Collections were made in villages, cities, and nearby natural environments by visually scanning artificial containers left outdoors and recovering larvae and pupae with a metal sieve. Adults were either collected directly in the field by aspirating females approaching the experimenter or emerged from field-collected larvae and pupae. For gene silencing experiments, adults emerged from field-collected larvae and pupae were used.

### Mosquito dissection and RNA extraction

For RNA expression profile of *Ae. albopictus* across developmental stages, pupa and larvae of the indicated instar (without differentiating sex) were homogenized for RNA extraction. For RNA expression profile of *Ae. albopictus* across developmental stages, adult mosquitoes aged 7 days after eclosion were subjected for total RNA extraction unless otherwise indicated. Female adult mosquitoes 3 days post injection of dsRNA were homogenized for RNA extraction and quantification of gene expression unless otherwise indicated. The mosquitoes and their tissues were homogenized and RNA was isolated using the Multisource RNA miniprep Kit (AP-MN-MS-RNA-250G, Axygen). cDNA was synthesized with Primescript RT reagent kit (RR037A, Takara) following the manufacturer’s instruction.

### Quantification of gene expression

Gene expression was quantified via real-time PCRs (qPCRs) using the LightCycler 480 SYBR Green I Master (04887352001, Roche) in the Quantagene q225 thermal cycler system (q225, Kubo technology). The primers used for this analysis are listed in Additional file 8: Table S2. The indicated gene expression was normalized against *Ae. albopictus* (*AALF010408*) or *Ae. aegypti* actin (*AAEL011197*).

### Gene silencing in mosquitoes

Double-strand RNA (dsRNA) for gene silencing was synthetized using MEGAscript T7 transcription kit (AM1334, Invitrogen). Mosquitoes used for gene silencing were adult females 0–5 days post-eclosion that had previously mated but not taken a bloodmeal. Female mosquitoes were anaesthetized with carbon dioxide (CO_2_) and was intro-thoracic microinjected with 1 μg per 300 nl of dsRNA using the Nanoject (3-000-207, Drummond). The injected mosquitoes were allowed to recover for 3 days under standard rearing conditions before subsequent behavior experiments. The gene silencing efficiency was evaluated via qPCR. The primers used for dsRNA synthesis and gene expression analysis are shown in Additional file 8: Table S2.

### Generation of ocularly blindfolded mosquitoes

Ocularly blindfolded mosquitoes were generated using methods previously described for *Drosophila melanogaster* [30–32]. Both eyes of female mosquitoes were painted with an opaque, black, and water-soluble acrylic paint (793, Marie’s). Specifically, mosquitoes were anesthetized with CO_2_, and a painting brush was used to carefully apply one coat of water diluted acrylic paint. Applying the paint into coverslips in the same way reduced the light flux down to 0.86% measured with a luminometer (AS-V10, Aicevoos). Mosquitoes with the pigment layer painted over eyes were double checked before the photobehavior with Y-maze assay with overhead LED illumination at 120 lux.

### Photobehavior assay of adult mosquitoes

Photobehavior assay of adult mosquitoes includes three assays tested mosquito preference over dark environment and visible light (Y-maze photobehavior assay, tube photobehavior assay, and shading assay) and one assay that tested mosquito preference over dark environment and UV light (photobehavior assay with blacklight). For photobehavior assay of adult mosquitoes without pretreatment, including Y-maze photobehavior assay, tube photobehavior assay, and shading assay, fifty mosquitoes were released in each individual repetition. For experiments with dsRNA injected or blindfolded mosquitoes, approximately twenty to forty mosquitoes were released in each individual repetition. One day prior to the behavior assays, mosquitoes were anaesthetized at 4 °C and the number of mosquitoes was counted before being transferred into new containers and maintained under standard conditions. The number of mosquitoes in dark environment was calculated as the total number of mosquitoes released subtracting the number of mosquitoes in illuminated environment and the number of mosquitoes that did not make a choice.

Except for testing the photobehavior of the *Aedes* and *Culex* mosquitoes across zeitgeber time, the photobehavior experiments with visible light were conducted during ZT1 to ZT5. The photobehavior of ocularly painted mosquitoes was tested simultaneously with control group, and the photobehavior of mosquitoes with *Opsin* knock down was tested simultaneously with the mock group injected dsRNA against *GFP*.

#### Y-maze photobehavior assay

This assay was used to test mosquito preference for illuminated versus dark environment (Fig. 1a). The custom-designed experiment apparatus comprised three chambers of quartz glass, including two arms of choice (5 cm in diameter and 25 cm in length) and an entrance (5 cm in diameter and 10 cm in length). One chamber of the two arms was wrapped with black tape, providing the mosquitoes with a choice of shaded (black box) versus light-exposed environment (overhead white light LED illumination at 120 lux). Light intensities across the Y-maze apparatus were measured with a luminometer (AS-V10, Aicevoos). Mosquitoes acclimated in the meshed release cage were released into the Y-maze, with most mosquito halt activity at 2 min post release (Additional file 2: Fig. S1c). Thus, distribution of mosquitoes within the two arms of the Y-maze was determined at 5 min post release. In order to prevent bias within the Y-maze, the two arms were routinely interchanged after each individual experiment. In each individual test, mosquitoes (approximately 55%) that did not make a choice by staying either in the release cage or in the central entrance chamber were excluded when calculating the preference index.

#### Tube photobehavior assay

This assay was modified from previously described assays [22, 37]. To investigate mosquito preference between illuminated and dark environment, the quartz glass tube (8 cm in diameter and 60 cm in length) was subdivided into four sections (two shaded areas and two light-exposed areas) with black tape (Fig. 1c). All areas were 15 cm in length. Overhead white light LED provided the light-exposed areas illumination at 120 lux. Light intensities across the tube apparatus were measured with a luminometer (AS-V10, Aicevoos). Mosquitoes were introduced to the end of the shaded or light-exposed end alternatively. Mosquitoes acclimated in the meshed cage were released, and most mosquitoes halt activity at 2 min post release (Additional file 2: Fig. S1d). Thus, distribution of mosquitoes of the tube was determined at 5 min post releasement. Mosquitoes (approximately 10%) that did not make a choice and stayed in the release cage were excluded.

To investigate mosquito preference over multiple illumination intensities, the quartz glass tube (8 cm in diameter and 60 cm in length) was subdivided into indicated equal sections, which were set in chambers with indicated illumination intensities (Additional file 2: Fig. S2). For each technical replicate, chambers were rearranged and mosquitoes were introduced to the indicated end. Light intensities across the tube apparatus of representative chamber arrangement were measured with a luminometer (AS-V10, Aicevoos). Mosquitoes were allowed to acclimate for 5 min in the meshed release cage and were given 5 min to make a choice.

#### Shading assay

To investigate mosquito preference between illuminated and dark environment, mosquitoes were release into illuminated quartz glass tube (8 cm in diameter and 60 cm in length) and were allowed to acclimated for 5 min. Then, the glass tube was carefully half-shaded without agitating the mosquitoes rested in. Mosquito preference index was calculated at 0 h, 0.5 h, 2 h, and 4 h post shading.

#### Photobehavior assay with blacklight

Tunable light source (CME-TLSX300F, Microenerg Technology) with narrow peak wavelength ranging from 300 to 2000 nm was used for UV light. Experiment apparatus of Y-maze assay was illuminated by UV light located 90 cm away of the end of one arm, with another arm wrapped in black tape (Fig. 1g). Light intensities around experiment apparatus were 400 μW/cm^2^ for 395 nm and 150 μW/cm^2^ for 345 nm UV light, as determined by a power meter (LS125+UVALED-X3, Shenzhen Linshang Technology).

### Larvae and pupae photobehavior assay

Measurements of larval and pupal photobehavior were made via plate assay and tray assay modified from a previous study [29]. Mixed-gender larvae and pupae of the indicated instar were used for photobehavior study. For plate assay, the plastic Petri plates (150 mm × 15 mm) were filled with water and placed upon a template which divided the plate into four equal quadrants to get two opposed light quadrants and two opposed shaded quadrants (Fig. 3a). LED light below the plate and template provided illumination at 120 lux. Fifty larvae were placed in the middle of the petri dish at the onset of testing and were given 5 min to make a choice. For tray assay, the transparent quartz glasses (200 mm × 50 mm × 50 mm) were filled with water and divided into four equal sections (two shaded areas and two light-exposed areas) with black paper (Fig. 3c). LED light below the tray provided illumination at 120 lux. Fifty larvae or pupae were placed in the middle of the petri dish at the onset of testing and were given 5 min to make a choice.

### Biting assay for host preference

This assay was used to test mosquito preference for host in illuminated *versus* host in dark environment (Fig. 4a) or hosts in environment with multiple illumination intensities (Fig. 4e). For host preference in illuminated *versus* host in dark environment, the experiment apparatus comprised three chambers (20 × 20 × 20 cm each) of acrylic board, including a chamber for mosquito releasing, a transparent chamber on one side, and a lightproof chamber on the other side (Fig. 4a). White LED lights provided overhead illumination. Female mosquitoes 5 days post microinjection of dsRNA were starved for 24 h before releasing into the middle of the chamber. For host preference over multiple illumination intensities, the experiment apparatus comprised five chambers (20 × 20 × 20 cm each) of acrylic board, including a chamber for mosquito releasing, one lightproof chamber, and three transparent chamber set in area with indicated illumination intensities (Fig. 4e).

Biting assay for *Aedes* was performed during ZT1 to ZT8, while biting assay for *Culex* was performed during ZT13 to ZT24 to maximize percentage of blood feeding. Mice (BALB/c strain, males, 6–8 weeks old) were anaesthetized and randomly assigned into chambers of indicated light intensity. Mosquitoes were allowed to fly through holes (10 cm diameter) between chambers and took a bloodmeal. The holes between chambers were sealed after 30 min, and mosquitoes were anaesthetized at 4 °C. The number of mosquitoes present at each chamber and the number of blood-fed mosquitoes were counted. Percentage of blood-fed mosquitoes was calculated as number of mosquitoes that blood-fed on the mice in the chamber with indicated illumination intensity divided by the total number of mosquitoes tested.

### Biting assay without host preference

This assay was used to test mosquito biting rate under indicated illumination. One day prior to the assay, mosquitoes were anaesthetized at 4 °C, and 20–25 female mosquitoes were transferred into transparent containers. After 24 h starvation, mosquitoes were subjected to biting assay under environment with indicated illumination. Anaesthetized mice were randomly assigned to the mesh top of the transparent containers (Additional file 2: Fig. S4a). Biting assay with four different illumination was performed simultaneously. After 30 min, mosquitoes were anaesthetized at 4 °C and the number of blood-fed mosquitoes were counted. Percentage of blood-fed mosquitoes was calculated as number of mosquitoes that fully engorged divided by the total number of mosquitoes tested.

### Statistical analysis

All data is presented as mean ± SEM. Statistical analysis and graphing were performed using GraphPad Prism 8 (Prism, La Jolla, CA, USA). Preference index was calculated as (*S* − *I*)/(*S* + *I*), where *S* denotes the number of mosquitoes that preferred the shaded chamber and *I* denotes the number of mosquitoes that preferred the illuminated chamber. The sum of *S* and *I* denotes the total number of mosquitoes that made a choice.

To test whether the preference indices represent photobehavior that statistically differs from chance, one sample *t* test was performed for data that met the assumptions of normality and Wilcoxon signed-rank test was performed for data with skewed distribution. Unpaired *t* test or Mann-Whitney test was performed to determine the significance of photobehavior between two groups. For unpaired *t* test, data were tested and met the assumptions of normality and homogeneity of variance. One-way ANOVA, followed by Tukey’s multiple comparisons test was performed to determine the significance of photobehavior among different groups that met the assumptions of normality. Kruskal-Wallis test, followed by Dunn’s multiple comparisons test, was performed for testing among different groups that does not met the assumptions of normality. Two-way ANOVA, followed by Sidak’s multiple comparisons test, was performed to determine the significance of photobehavior between two groups for a consecutive of 3 days. Two-way ANOVA, followed by Tukey’s multiple comparisons test, was performed to determine the significance of biting behavior between control and blindfolded or dsRNA treatment group. Different lowercase letters indicate significant difference. Experimental groups denoted by “ab” are not significantly different from either “a” or “b” groups. Sample size (*n*) of each experiment was indicated in the figure legends, and we typically performed no less than three independent biological replicates (N). Confidence level to reject the null hypothesis that the mean of the data set is chance or two groups have the same means was indicated by *P* value. Statistical significance was at the level of *P* < 0.05, specifically ns: not significant, *P < 0.05, **P < 0.01, *** *P* < 0.001, **** *P* < 0.0001.

## Supplementary Information

**Additional file 1: Video S1.** Photopreference of *Ae. aegypti* adults with Y-maze assay (related to Fig. 1b). This video shows the photopreference of female mosquitoes tested with Y-maze assay which was slightly modified to facilitate video shotting. In the manuscript, choosing arm of the dark environment was completely wrapped in black tape to minimize light transmittance, and overhead LED provided illumination. In this video, screen light below the Y-maze provided illumination and cardboard paper shaded light for the choosing arm of the dark environment. Two cameras (canon 80D) recorded video simultaneously under different exposure settings. The video was sped up 4x times.

**Additional file 2: Fig. S1.** Photonegative behavior of adult mosquitoes, related to Fig. 1. (a-b) Y-maze assay with host cues. (a) Assay schematic. (b) Preference between illuminated and shaded environment. *n* = 150 females per species. The data are presented as mean ± SEM. Photobehavior were analyzed using one sample *t* test. Rejection of the null hypothesis that the mean of the data set is chance: ****p < 0.0001. (c-d) Mosquito activity post release. Upper panel: assay schematic. The assay was conducted with Y-maze or tube with the entire apparatus under 120 lux. Lower panels: mosquito activity in Y-maze assay (c) or tube assay (d) post release. Three sample activity patterns from a pool of 8-12 *Ae. albopictus* individuals is shown. *n* in the figure denotes the total number of mosquitoes tested. **Fig. S2.** Schematic presentation of photopreference assay, related to Fig. 2. (a) Schematic presentation of binary photopreference assay between 0 lux and 15 lux. (b) Schematic presentation of binary photopreference assay between 15 lux and 150 lux. (c) Schematic presentation of binary photopreference assay between 150 lux and 1500 lux. (d) Schematic presentation of trinary photopreference assay with 0 lux, 15 lux and 150 lux (e). Schematic presentation of trinary photopreference assay with 15 lux, 150 lux and 1500 lux. (f) Mosquitoes were allowed to make a choice from environment of 0 lux, 15 lux, 150 lux and 1500 lux. (a-f) Arrow head indicates where mosquitoes were released. **Fig. S3.** Photobehavior of forth instar larvae and pupae of *Ae. albopictus* mosquito, related to Fig. 3. (a-c) Plate assay. (a) Schematic indicating how the quadrants were annotated. (b-c) Percentage of forth instar larvae (b) or pupae (c) of *Ae. albopictus* that preferred the indicated region. *n* = 200 larvae or pupae. (d-f) Tray assay. (d) Schematic indicating how the regions were annotated. (e-f) Percentage of forth instar larvae (e) or pupae (f) of *Ae. albopictus* that preferred the indicated region. *n* = 200 larvae or pupae. (b-c, e-f) Dots represent the percentage of *Ae. albopictus* that preferred the indicated region in individual repeats. For each group, four biological replicates with four technical replicates were performed. Total number of animals tested are indicated. (b, e) One-way ANOVA, followed by Tukey's multiple comparisons test was performed for testing among different groups. (c, f) Kruskal-Wallis test, followed by Dunn's multiple comparisons test was performed for testing among different groups. Different lowercase letters indicate significantly different. **Fig. S4.** Biting behavior of female mosquitoes under indicated illumination intensities, related to Fig. 4. (a) Schematic of biting assay under indicated illumination intensities without preference between hosts. (b-d) Percent of *Ae. aegypti* (b), *Ae. albopictus* (c) and *Cx. quinquefasciatus* (d) that bitted hosts under environment with indicated illumination intensities. Data are presented as mean ± SEM. Each dot indicates the blood feeding rate of one biological replicate. *n* in the figure denotes the total number of mosquitoes tested. The total number of mosquitoes fully engorged relative to the total number of mosquitoes tested is shown at the top of each column. One-way ANOVA test performed for testing among different groups, which showed no significant difference. **Fig. S5.** Phylogenetic tree based on protein sequences of opsins from five species of Dipteran. generated with neighbor-joining tree, related to Fig. 5. Neighbor-joining tree constructed using MEGA5 with Poisson model+uniform rates*. Aalb* denotes *Ae. albopictus*, *Aaeg* denotes *Ae. aegypti*, *Cqui* denotes *Cx. quinquefasciatus*, *Dmel* denotes *D. melanogaster*. The bar indicates phylogenetic distance. **Fig. S6.** Knocking down efficiency and biting behavior of *opsin1*-silenced *Ae. albopictus*, related to Figs. 5 and 6. (a-d) Expression of *Aalb*Opsins upon microinjection of dsRNA. *AalbOpsin1* (a), *AalbOpsin2* (b), *AalbOpsin8* (c) and *AalbOpsin9* (d) expression three days post microinjection of *AalbOpsin1* dsRNA. *n* = 10 mosquitoes pre group. (e-h) Expression of *Aalb*Opsins upon microinjection of dsRNA. *AalbOpsin1* (e), *AalbOpsin2* (f), *AalbOpsin8* (g) and *AalbOpsin9* (h) expression three days post microinjection of *AalbOpsin2* dsRNA. *n* = 10 mosquitoes pre group. (i) Expression of Opsins upon microinjection of dsRNA. *AalbOpsin8* expression three days post microinjection of *AalbOpsin8* dsRNA. *n* = 10 mosquitoes pre group. (j) Expression of Opsins upon microinjection of dsRNA. *AalbOpsin9* expression three days post microinjection of *AalbOpsin9* dsRNA. *n* = 10 mosquitoes pre group. (k) Sequence identities between *Aalb*Opsins in percentage. Pairwise comparison of amino acid sequences of opsins of *Ae. albopictus*. Sequence similarity was presented with heatmap. (l) Sequence identities between *Aalb*Opsins and *Aaeg*Opsins in percentage. Pairwise comparison of amino acid sequences of *Aalb*Opsins and *Aaeg*Opsins. Opsins of *Ae. albopictus* share 90.74% to 97.59% similarity with the corresponding opsins of *Ae. aegypti*. Sequence similarity was presented with heatmap. (m) *Aaeg*Opsin1 expression three days post microinjection of dsRNA. Expression levels of *Aaeg*Opsin1 were normalized against *Ae. aegypti actin* (*AAEL011197*). *n* = 10 females per group. (n) Photobehavior of *opsin1*-silenced *Ae. albopictus*. Three days post thoracic inoculation of dsRNA, biting preference between hosts in illuminated and shaded environment was assessed (ds*GFP*, *n* = 113 females; ds*Aalb*Opisn1^#1^, *n* = 111 females). The data are presented as mean ± SEM. Two-way ANOVA, followed by Tukey's multiple comparisons test was performed for testing among different groups. Different lowercase letters indicate significantly different. (a-j, m) The data are presented as mean ± SEM, with each dot represents one mosquito. (a-j) Expression levels of *AalbOpsin* genes were normalized against *Ae. albopictus actin* (*AALF010408*). (a-c, f, h, j, m) Mann-Whitney test was performed for testing between two groups. (d-e, g, i) Unpaired *t* test was performed for testing among different groups. Rejection of the null hypothesis that two groups have the same mean: *p < 0.05, **p < 0.01, ***p < 0.001, ****p < 0.0001, ns: not significant. **Fig. S7.** Field collection of *Ae. albopictus* in three districts and photobehavior of field-collected larval and pupae. (a) *Ae. albopictus* were collected from three sites in three districts. One site in district A (112°56′E, 28°11′N), one site in district B (112°1′E, 27°43′N) and one site in district C (111°56′E, 28°53′N). (b) Pictures of typical collection sites. (c) Tray assay to test photobehavior of field-collected larvae and pupae. Preference index of field-collected *Ae. albopictus* between illuminated and shaded environment (*n* = 150 larvae or pupae per group). Data are presented as mean ± SEM. Photobehavior were analyzed using one sample *t* test or Wilcoxon signed-rank test. *p < 0.05, ***p < 0.001, ****p < 0.001.

**Additional file 3: Video S2.** Photopreference of *Ae. aegypti* adults with tube assay (related to Fig. 1d). This video shows the photopreference of female mosquitoes tested with tube assay which was slightly modified to facilitate video shotting. In the study, choosing arm of the dark environment was completely wrapped in black tape to minimize light transmittance and overhead LED provided illumination for choosing arm of the illuminated environment. In this video, LED light below the tube provided illumination and black paper shaded light for the choosing arm of the dark environment. Two cameras (canon 80D) recorded video simultaneously under different exposure settings. The video was sped up 4x times.

**Additional file 4: Video S3.** Photopreference of *Ae. aegypti* larvae with plate assay (related to Fig. 3b). This video shows the photopreference of fourth instar larvae and pupae of *Aedes aegypti* tested with plate assay. In this video, screen light below the plate provided illumination. Two cameras (canon 80D) recorded video simultaneously under different exposure settings. The video was sped up 4x times.

**Additional file 5: Video S4.** Photopreference of *Ae. aegypti* pupae with plate assay (related to Fig. 3b). This video shows the photopreference of fourth instar larvae and pupae of *Aedes aegypti* tested with plate assay. In this video, screen light below the plate provided illumination. Two cameras (canon 80D) recorded video simultaneously under different exposure settings. The video was sped up 4x times.

**Additional file 6: Video S5.** Photopreference of *Ae. aegypti* larvae with tray assay (related to Fig. 3d). This video shows the photopreference of fourth instar larvae and pupae of *Aedes aegypti* tested with tray assay. In this video, screen light below the tray provided illumination. Two cameras recorded video simultaneously under different exposure settings. The video was sped up 4x times.

**Additional file 7: Video S6.** Photopreference of *Ae. aegypti* pupae with tray assay (related to Fig. 3d). This video shows the photopreference of fourth instar larvae and pupae of *Aedes aegypti* tested with tray assay. In this video, screen light below the tray provided illumination. Two cameras (canon 80D) recorded video simultaneously under different exposure settings. The video was sped up 4x times.

**Additional file 8: Table S1.** Summary of *Ae. albopictus*, *Ae. aegypti* and *Cx. quinquefasciatus opsin* genes. **Table S2.** Primers for qPCR and dsRNA synthesis.

## Data Availability

The exact number of mosquitoes that chose illumination or dark environment in each individual experiment, light intensities across the experiment apparatus, and a table summary of Shapiro-Wilk test checking whether the data meet normality assumptions are available in the Mendeley Data repository [45]. Further information and requests for resources and reagents should be directed to and will be fulfilled by the lead contact, Pa Wu (wupa@hunnu.edu.cn).

## Abbreviations

PI: Preference index
GPCR: G protein-coupled receptor
ZT: Zeitgeber time
RNAi: RNA interference
UV: Ultraviolet
LED: Light-emitting diode
GFP: Green fluorescent protein
qPCR: Quantitative polymerase chain reaction
