# Supplementary figures and images for: Opsin1 regulates light-evoked avoidance behavior in Aedes albopictus
Source: BMC Biol. 2022 May 13;20:110. doi: 10.1186/s12915-022-01308-0 (PMC9103082; doi:10.1186/s12915-022-01308-0)

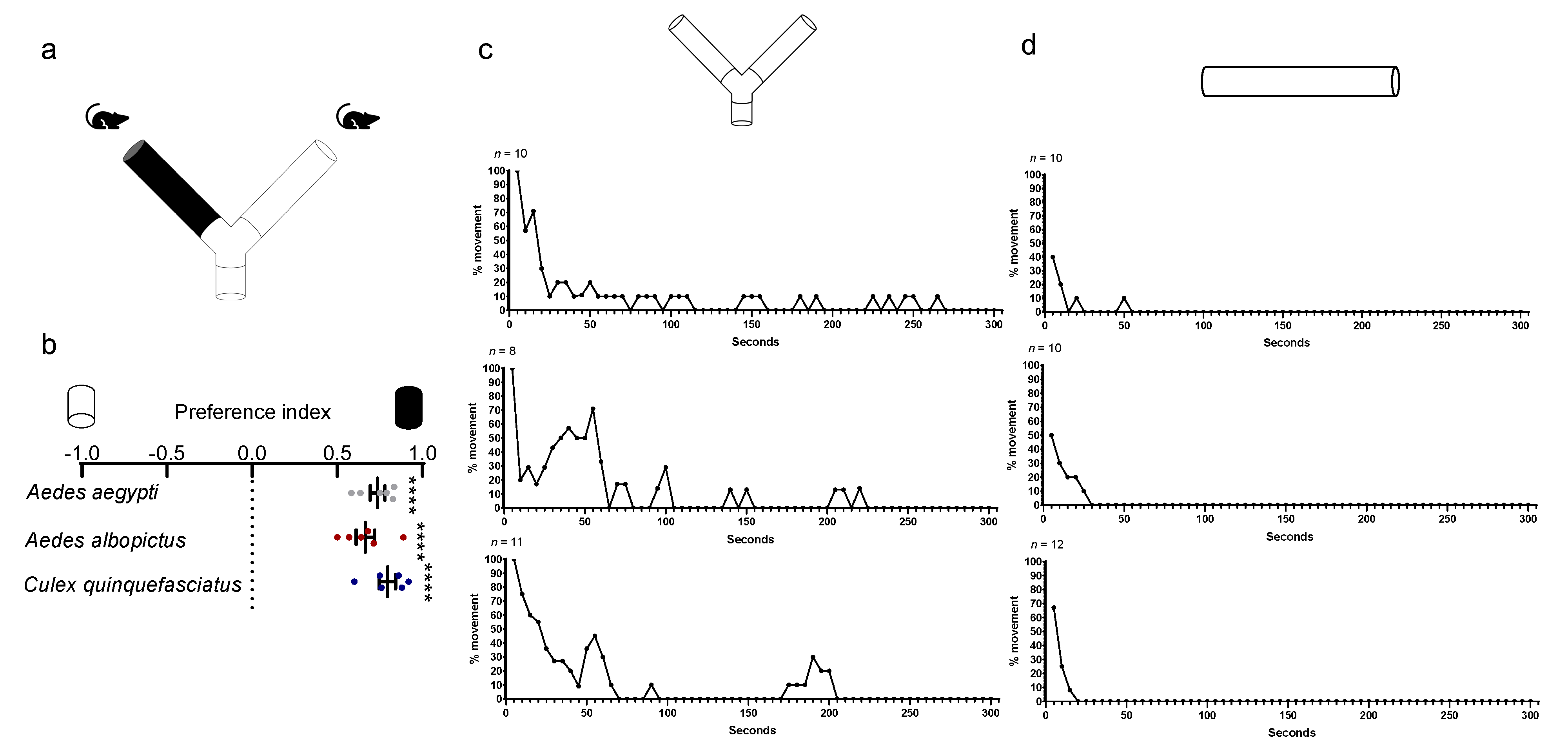

Supplement: Supplementary file 2 — Additional file 2: Fig. S1. Photonegative behavior of adult mosquitoes, related to Fig. 1. (a-b) Y-maze assay with host cues. (a) Assay schematic. (b) Preference between illuminated and shaded environment. n = 150 females per species. The data are presented as mean ± SEM. Photobehavior were analyzed using one sample t test. Rejection of the null hypothesis that the mean of the data set is chance: ****p < 0.0001. (c-d) Mosquito activity post release. Upper panel: assay schematic. The assay was conducted with Y-maze or tube with the entire apparatus under 120 lux. Lower panels: mosquito activity in Y-maze assay (c) or tube assay (d) post release. Three sample activity patterns from a pool of 8-12 Ae. albopictus individuals is shown. n in the figure denotes the total number of mosquitoes tested. Fig. S2. Schematic presentation of photopreference assay, related to Fig. 2. (a) Schematic presentation of binary photopreference assay between 0 lux and 15 lux. (b) Schematic presentation of binary photopreference assay between 15 lux and 150 lux. (c) Schematic presentation of binary photopreference assay between 150 lux and 1500 lux. (d) Schematic presentation of trinary photopreference assay with 0 lux, 15 lux and 150 lux (e). Schematic presentation of trinary photopreference assay with 15 lux, 150 lux and 1500 lux. (f) Mosquitoes were allowed to make a choice from environment of 0 lux, 15 lux, 150 lux and 1500 lux. (a-f) Arrow head indicates where mosquitoes were released. Fig. S3. Photobehavior of forth instar larvae and pupae of Ae. albopictus mosquito, related to Fig. 3. (a-c) Plate assay. (a) Schematic indicating how the quadrants were annotated. (b-c) Percentage of forth instar larvae (b) or pupae (c) of Ae. albopictus that preferred the indicated region. n = 200 larvae or pupae. (d-f) Tray assay. (d) Schematic indicating how the regions were annotated. (e-f) Percentage of forth instar larvae (e) or pupae (f) of Ae. albopictus that preferred the indicated region. n = 2 [file 12915_2022_1308_MOESM2_ESM.zip › S1_Fig.tiff]

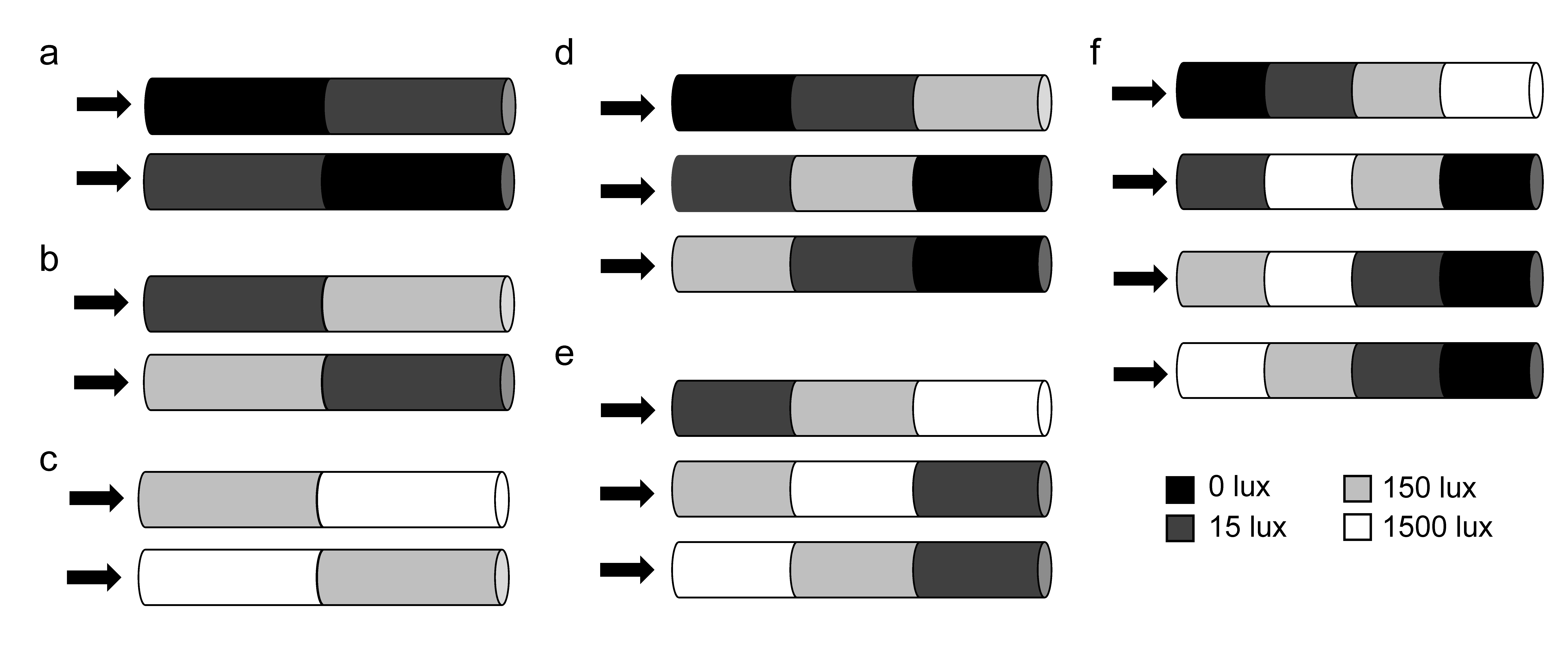

Supplement: Supplementary file 2 — Additional file 2: Fig. S1. Photonegative behavior of adult mosquitoes, related to Fig. 1. (a-b) Y-maze assay with host cues. (a) Assay schematic. (b) Preference between illuminated and shaded environment. n = 150 females per species. The data are presented as mean ± SEM. Photobehavior were analyzed using one sample t test. Rejection of the null hypothesis that the mean of the data set is chance: ****p < 0.0001. (c-d) Mosquito activity post release. Upper panel: assay schematic. The assay was conducted with Y-maze or tube with the entire apparatus under 120 lux. Lower panels: mosquito activity in Y-maze assay (c) or tube assay (d) post release. Three sample activity patterns from a pool of 8-12 Ae. albopictus individuals is shown. n in the figure denotes the total number of mosquitoes tested. Fig. S2. Schematic presentation of photopreference assay, related to Fig. 2. (a) Schematic presentation of binary photopreference assay between 0 lux and 15 lux. (b) Schematic presentation of binary photopreference assay between 15 lux and 150 lux. (c) Schematic presentation of binary photopreference assay between 150 lux and 1500 lux. (d) Schematic presentation of trinary photopreference assay with 0 lux, 15 lux and 150 lux (e). Schematic presentation of trinary photopreference assay with 15 lux, 150 lux and 1500 lux. (f) Mosquitoes were allowed to make a choice from environment of 0 lux, 15 lux, 150 lux and 1500 lux. (a-f) Arrow head indicates where mosquitoes were released. Fig. S3. Photobehavior of forth instar larvae and pupae of Ae. albopictus mosquito, related to Fig. 3. (a-c) Plate assay. (a) Schematic indicating how the quadrants were annotated. (b-c) Percentage of forth instar larvae (b) or pupae (c) of Ae. albopictus that preferred the indicated region. n = 200 larvae or pupae. (d-f) Tray assay. (d) Schematic indicating how the regions were annotated. (e-f) Percentage of forth instar larvae (e) or pupae (f) of Ae. albopictus that preferred the indicated region. n = 2 [file 12915_2022_1308_MOESM2_ESM.zip › S2_Fig.tiff]

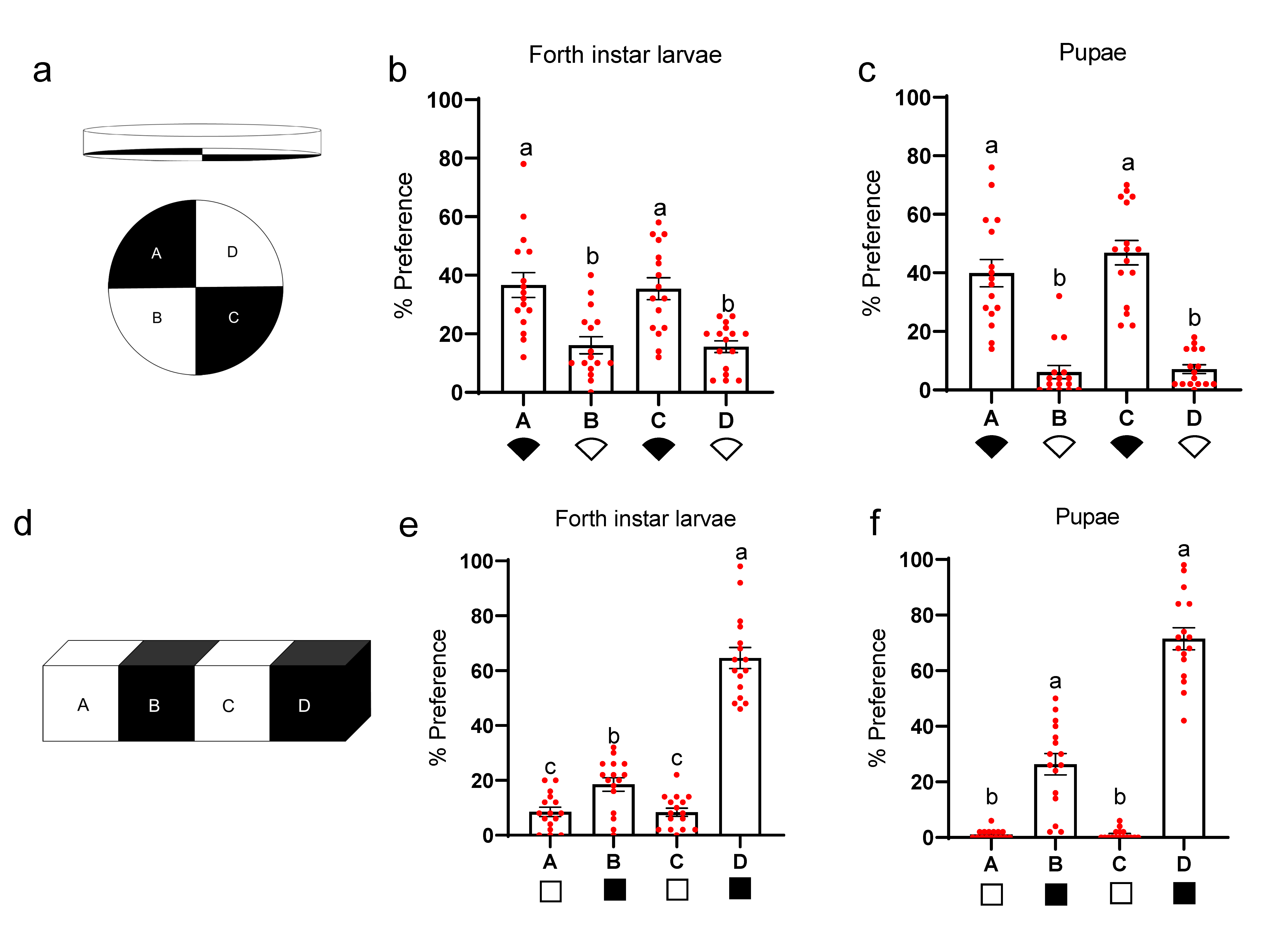

Supplement: Supplementary file 2 — Additional file 2: Fig. S1. Photonegative behavior of adult mosquitoes, related to Fig. 1. (a-b) Y-maze assay with host cues. (a) Assay schematic. (b) Preference between illuminated and shaded environment. n = 150 females per species. The data are presented as mean ± SEM. Photobehavior were analyzed using one sample t test. Rejection of the null hypothesis that the mean of the data set is chance: ****p < 0.0001. (c-d) Mosquito activity post release. Upper panel: assay schematic. The assay was conducted with Y-maze or tube with the entire apparatus under 120 lux. Lower panels: mosquito activity in Y-maze assay (c) or tube assay (d) post release. Three sample activity patterns from a pool of 8-12 Ae. albopictus individuals is shown. n in the figure denotes the total number of mosquitoes tested. Fig. S2. Schematic presentation of photopreference assay, related to Fig. 2. (a) Schematic presentation of binary photopreference assay between 0 lux and 15 lux. (b) Schematic presentation of binary photopreference assay between 15 lux and 150 lux. (c) Schematic presentation of binary photopreference assay between 150 lux and 1500 lux. (d) Schematic presentation of trinary photopreference assay with 0 lux, 15 lux and 150 lux (e). Schematic presentation of trinary photopreference assay with 15 lux, 150 lux and 1500 lux. (f) Mosquitoes were allowed to make a choice from environment of 0 lux, 15 lux, 150 lux and 1500 lux. (a-f) Arrow head indicates where mosquitoes were released. Fig. S3. Photobehavior of forth instar larvae and pupae of Ae. albopictus mosquito, related to Fig. 3. (a-c) Plate assay. (a) Schematic indicating how the quadrants were annotated. (b-c) Percentage of forth instar larvae (b) or pupae (c) of Ae. albopictus that preferred the indicated region. n = 200 larvae or pupae. (d-f) Tray assay. (d) Schematic indicating how the regions were annotated. (e-f) Percentage of forth instar larvae (e) or pupae (f) of Ae. albopictus that preferred the indicated region. n = 2 [file 12915_2022_1308_MOESM2_ESM.zip › S3_Fig.tiff]

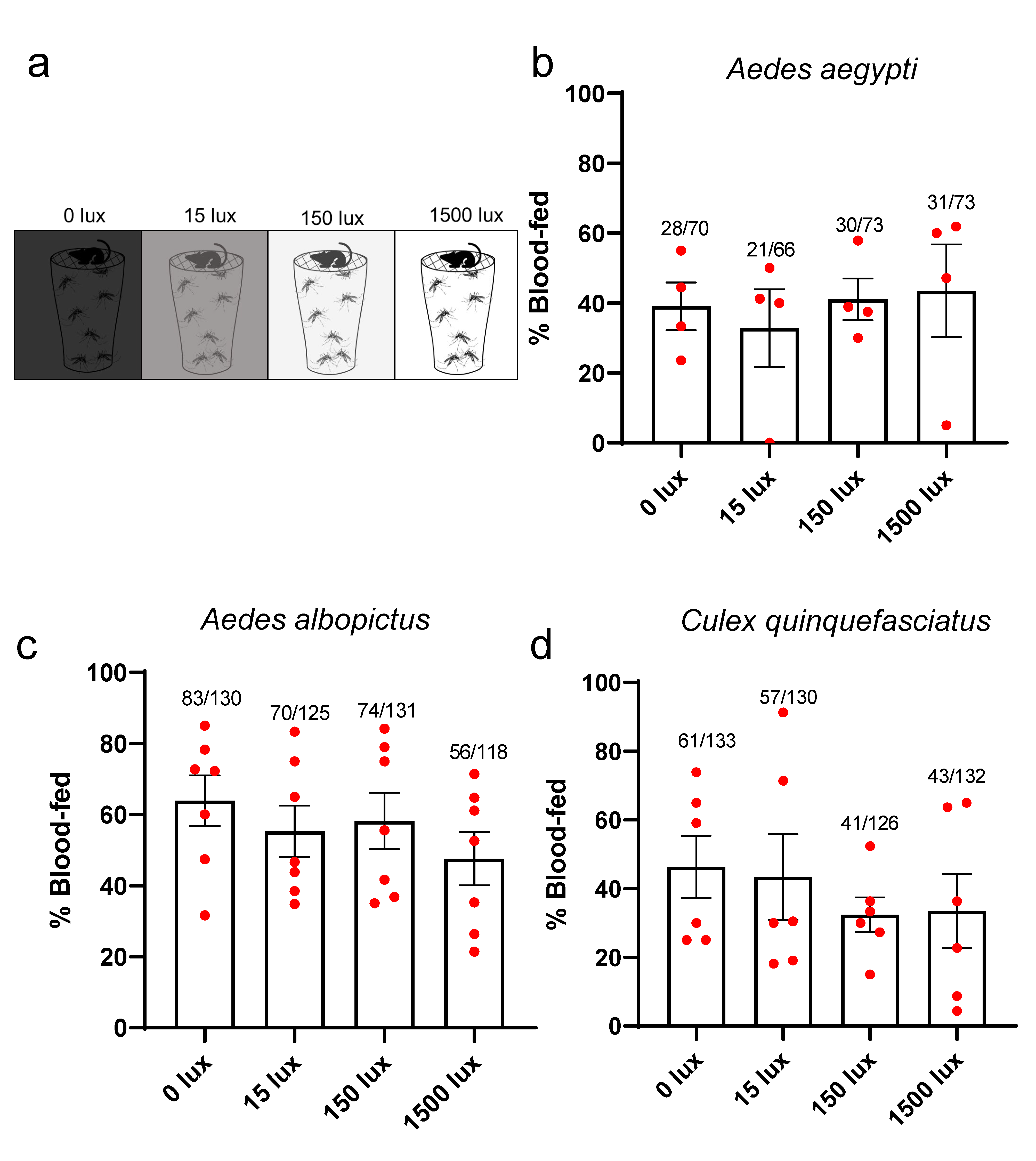

Supplement: Supplementary file 2 — Additional file 2: Fig. S1. Photonegative behavior of adult mosquitoes, related to Fig. 1. (a-b) Y-maze assay with host cues. (a) Assay schematic. (b) Preference between illuminated and shaded environment. n = 150 females per species. The data are presented as mean ± SEM. Photobehavior were analyzed using one sample t test. Rejection of the null hypothesis that the mean of the data set is chance: ****p < 0.0001. (c-d) Mosquito activity post release. Upper panel: assay schematic. The assay was conducted with Y-maze or tube with the entire apparatus under 120 lux. Lower panels: mosquito activity in Y-maze assay (c) or tube assay (d) post release. Three sample activity patterns from a pool of 8-12 Ae. albopictus individuals is shown. n in the figure denotes the total number of mosquitoes tested. Fig. S2. Schematic presentation of photopreference assay, related to Fig. 2. (a) Schematic presentation of binary photopreference assay between 0 lux and 15 lux. (b) Schematic presentation of binary photopreference assay between 15 lux and 150 lux. (c) Schematic presentation of binary photopreference assay between 150 lux and 1500 lux. (d) Schematic presentation of trinary photopreference assay with 0 lux, 15 lux and 150 lux (e). Schematic presentation of trinary photopreference assay with 15 lux, 150 lux and 1500 lux. (f) Mosquitoes were allowed to make a choice from environment of 0 lux, 15 lux, 150 lux and 1500 lux. (a-f) Arrow head indicates where mosquitoes were released. Fig. S3. Photobehavior of forth instar larvae and pupae of Ae. albopictus mosquito, related to Fig. 3. (a-c) Plate assay. (a) Schematic indicating how the quadrants were annotated. (b-c) Percentage of forth instar larvae (b) or pupae (c) of Ae. albopictus that preferred the indicated region. n = 200 larvae or pupae. (d-f) Tray assay. (d) Schematic indicating how the regions were annotated. (e-f) Percentage of forth instar larvae (e) or pupae (f) of Ae. albopictus that preferred the indicated region. n = 2 [file 12915_2022_1308_MOESM2_ESM.zip › S4_Fig.tiff]

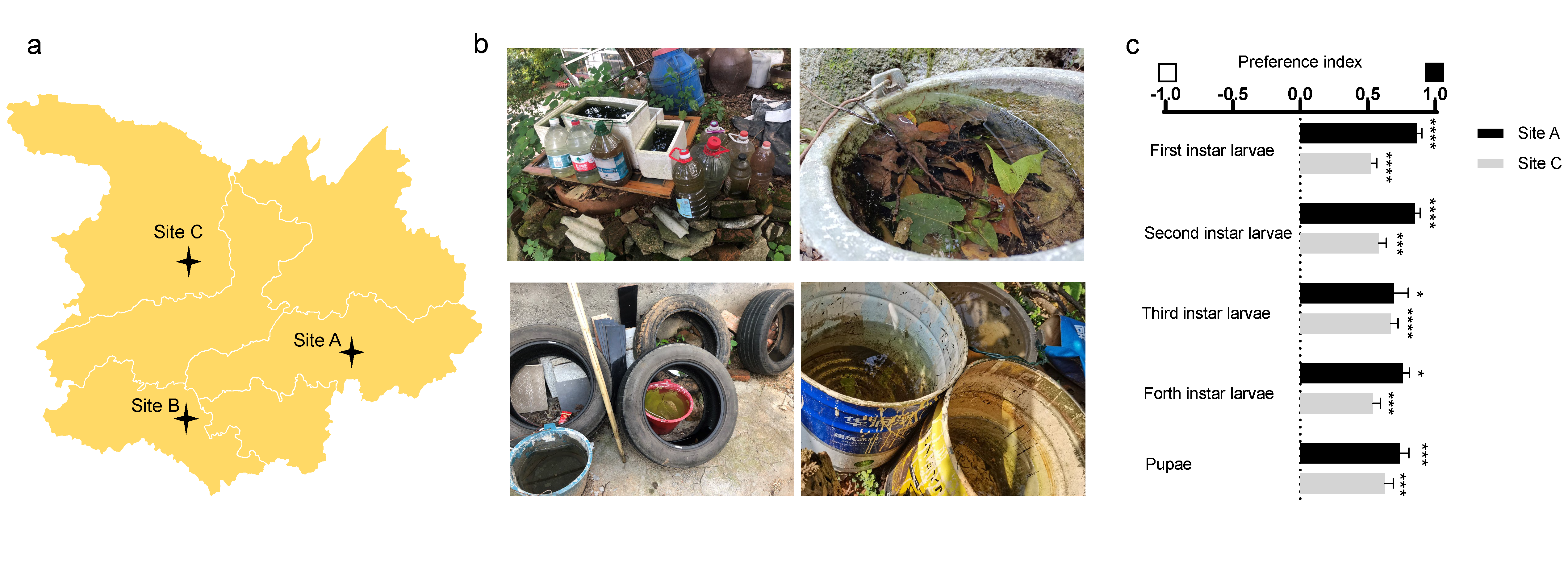

Supplement: Supplementary file 2 — Additional file 2: Fig. S1. Photonegative behavior of adult mosquitoes, related to Fig. 1. (a-b) Y-maze assay with host cues. (a) Assay schematic. (b) Preference between illuminated and shaded environment. n = 150 females per species. The data are presented as mean ± SEM. Photobehavior were analyzed using one sample t test. Rejection of the null hypothesis that the mean of the data set is chance: ****p < 0.0001. (c-d) Mosquito activity post release. Upper panel: assay schematic. The assay was conducted with Y-maze or tube with the entire apparatus under 120 lux. Lower panels: mosquito activity in Y-maze assay (c) or tube assay (d) post release. Three sample activity patterns from a pool of 8-12 Ae. albopictus individuals is shown. n in the figure denotes the total number of mosquitoes tested. Fig. S2. Schematic presentation of photopreference assay, related to Fig. 2. (a) Schematic presentation of binary photopreference assay between 0 lux and 15 lux. (b) Schematic presentation of binary photopreference assay between 15 lux and 150 lux. (c) Schematic presentation of binary photopreference assay between 150 lux and 1500 lux. (d) Schematic presentation of trinary photopreference assay with 0 lux, 15 lux and 150 lux (e). Schematic presentation of trinary photopreference assay with 15 lux, 150 lux and 1500 lux. (f) Mosquitoes were allowed to make a choice from environment of 0 lux, 15 lux, 150 lux and 1500 lux. (a-f) Arrow head indicates where mosquitoes were released. Fig. S3. Photobehavior of forth instar larvae and pupae of Ae. albopictus mosquito, related to Fig. 3. (a-c) Plate assay. (a) Schematic indicating how the quadrants were annotated. (b-c) Percentage of forth instar larvae (b) or pupae (c) of Ae. albopictus that preferred the indicated region. n = 200 larvae or pupae. (d-f) Tray assay. (d) Schematic indicating how the regions were annotated. (e-f) Percentage of forth instar larvae (e) or pupae (f) of Ae. albopictus that preferred the indicated region. n = 2 [file 12915_2022_1308_MOESM2_ESM.zip › S7_Fig.tiff]
