# Supplementary material for: Opsin1 regulates light-evoked avoidance behavior in Aedes albopictus
Source: BMC Biol. 2022 May 13;20:110. doi: 10.1186/s12915-022-01308-0 (PMC9103082; doi:10.1186/s12915-022-01308-0)
Supplement: Supplementary file 8 — Additional file 8: Table S1. Summary of Ae. albopictus, Ae. aegypti and Cx. quinquefasciatus opsin genes. Table S2. Primers for qPCR and dsRNA synthesis. [file 12915_2022_1308_MOESM8_ESM.zip › S1_Table.docx]

**S1 Table. Summary of *Aedes albopictus*, *Aedes aegypti* and *Culex quinquefasciatus* *opsin* genes.**

| **Sequence** | **Gene** | **NCBI accession no.** | **VectorBase gene ID** | **Protein** |
| --- | --- | --- | --- | --- |
| ***Ae.aegypti*** | *Aaeg*Opsin1 | XM_001651947.2 | AAEL006498 | XP_001651997.1 |
|  | *Aaeg*Opsin2 | XM_001657569.2 | AAEL006259 | XP_001657619.2 |
|  | *Aaeg*Opsin3 | XM_001651948.2 | AAEL006484 | XP_001651998.1 |
|  | *Aaeg*Opsin4 | XM_001651116.3 | AAEL005621 | XP_001651166.1 |
|  | *Aaeg*Opsin5 | XM_001651117.2 | AAEL005625 | XP_001651167.1 |
|  | *Aaeg*Opsin7 | XM_001652675.2 | AAEL007389 | XP_001652725.2 |
|  | *Aaeg*Opsin8 | XM_001653816.2 | AAEL009615 | XP_021698798.1 |
|  | *Aaeg*Opsin9 | XM_001662932.2 | AAEL003035 | XP_001662982.2 |
|  | *Aaeg*Opsin10 | XM_001650694.1 | AAEL005322 | XP_021702394.1 |
|  | *Aaeg*Opsin12 | XM_001650752.2 | AAEL005373 | XP_001650802.3 |
| ***Ae.albpictus*** | *Aalb*Opsin1 | XM_019696345.2 | LOC109421796 | XP_019551890.1 |
|  | *Aalb*Opsin2 | XR_003896737.1 | LOC109399710 | XP_019527761.1 |
|  | *Aalb*Opsin3 | XM_019696344.2 | LOC109421795 | XP_019553172.2 |
|  | *Aalb*Opsin4 | XM_019692389.2 | LOC109418231 | XP_019547934.2 |
|  | *Aalb*Opsin5 | XM_019703662.2 | LOC109428005 | XP_019559207.2 |
|  | *Aalb*Opsin7 | XM_019682214.2 | LOC109408846 | XP_019537759.2 |
|  | *Aalb*Opsin8 | XM_029874483.1 | LOC109398017 | XP_029730343.1 |
|  | *Aalb*Opsin9 | XM_029861840.1 | LOC115260681 | XP_029717700.1 |
|  | *Aalb*Opsin10 | XM_029870013.1 | LOC115265343 | XP_029725873.1 |
|  | *Aalb*Opsin12 | XM_029867276.1 | LOC115263972 | XP_029723136.1 |
| ***Cx. quinquefasciatus*** | *Cqui*Opsin1 | XM_001845645.2 | CPIJ004067 | XP_001845697.1 |
|  | *Cqui*Opsin2 | XM_001845589.1 | CPIJ005000 | XP_039440751.1 |
|  | *Cqui*Opsin3 | XM_001851105.2 | CPIJ009246 | XP_001851157.1 |
|  | *Cqui*Opsin4 | XM_001861603.1 | CPIJ011419 | XP_038116053.1 |
|  | *Cqui*Opsin5 | XM_001862130.2 | CPIJ012052 | XP_001862165.2 |
|  | *Cqui*Opsin6 | XM_001862163.2 | CPIJ011571 | XP_039433756.1 |
|  | *Cqui*Opsin7 | XM_001862165.2 | CPIJ011573 | XP_039433779.1 |
|  | *Cqui*Opsin8 | XM_001862166.1 | CPIJ011574 | XP_001862203.1 |
|  | *Cqui*Opsin9 | XM_001862168.2 | CPIJ011576 | XP_001862203.1 |
|  | *Cqui*Opsin10 | XM_001863451.1 | CPIJ013056 | XP_038114810.1 |
|  | *Cqui*Opsin11 | XM_001863511.2 | CPIJ013408 | XP_001863546.1 |
|  | *Cqui*Opsin12 | XM_001864516.1 | CPIJ014334 | XP_038121728.1 |
|  | *Cqui*Opsin13 | XM_001870251.2 | CPIJ020021 | XP_001870286.1 |
