# Supplementary material for: Opsin1 regulates light-evoked avoidance behavior in Aedes albopictus
Source: BMC Biol. 2022 May 13;20:110. doi: 10.1186/s12915-022-01308-0 (PMC9103082; doi:10.1186/s12915-022-01308-0)
Supplement: Supplementary file 8 — Additional file 8: Table S1. Summary of Ae. albopictus, Ae. aegypti and Cx. quinquefasciatus opsin genes. Table S2. Primers for qPCR and dsRNA synthesis. [file 12915_2022_1308_MOESM8_ESM.zip › S2_Table.docx]

**S2 Table. Primers for qPCR and dsRNA synthesis.**

| **Primers for double-strand RNA synthesis** | **Forward Primer** | **Reverse Primer** |
| --- | --- | --- |
| dsRNA GFP | TAATACGACTCACTATAGGGCAACGGTGTGGACTTTGAC | TAATACGACTCACTATAGGGCGGCTGGTTCTTCAGATAGTT |
| dsRNA *Aalb*Opsin1^#1^ | TAATACGACTCACTATAGGGCCGCATTGGAATCAGTTC | TAATACGACTCACTATAGGGCCAGGTGGTAGTAAGGTAATCG |
| dsRNA *Aalb*Opsin2 | TAATACGACTCACTATAGGGACTGTCCGGAGGAGAAGACA | TAATACGACTCACTATAGGGATGGGGCCAAAGTCCAGAAG |
| dsRNA *Aalb*Opsin8 | TAATACGACTCACTATAGGGCAACTTCACGGCTGTGCTTC | TAATACGACTCACTATAGGGGAAGCGTCCCCAGATTTCCA |
| dsRNA *Aalb*Opsin9 | TAATACGACTCACTATAGGGCTGATAAACGAAACCGATGC | TAATACGACTCACTATAGGGCACAGCCAGGTGATGAAA |
| dsRNA *Aalb*Opsin1^#2^ | TAATACGACTCACTATAGGGGCTACACTGAACACGCCTT | TAATACGACTCACTATAGGGGTATCGGTCAAACGCAATC |
| dsRNA *Aalb*Opsin1^#3^ | TAATACGACTCACTATAGGGCGCCATCTTTGTCTACTGG | TAATACGACTCACTATAGGGTTATGCCTTCTCGTCGGT |
| dsRNA *Aaeg*Opsin1 | TAATACGACTCACTATAGGGAGTTCCCACCGATGAATCC | TAATACGACTCACTATAGGGGGCGTAAACGATGATGTAGG |
| **Primers for SYBR Green RT-qPCR** | **Forward Primer** | **Reverse Primer** |
| *Aalb*Opsin1 (RT-qPCR for ds*Aalb*Opsin1^#1^ and ds*Aalb*Opsin1^#3^) | ACCGCTGATAGAGAATCGAGAG | TTGTCCACCACGGTCATGTT |
| *Aalb*Opsin1 (RT-qPCR for ds*Aalb*Opsin1^#2^) | ATCGTCTACGCCATCTTTG | CGAAACTGCCTTCAGGAT |
| *Aalb*Opsin2 | TGAAGTAGTTCGTTCGCCGT | AGGCTGCCATTGTCTTCTCC |
| *Aalb*Opsin3 | AAGTGTTTTAAGCGTCGCGG | GGACCTTATCCACCACGGTC |
| *Aalb*Opsin4 | GTTTGGATCACTGTTCGGATGC | GTTGTTGGTTAGAGGCTTGCCG |
| *Aalb*Opsin5 | TTTGGATCACTGTTCGGCTGT | TTGTTAGTCAGAGGCTTACCG |
| *Aalb*Opsin7 | TCGTGTACGGCATCAGCCAT | CACCACTAACTGCAGACATCCC |
| *Aalb*Opsin8 | GCTTCCCCTCATTTCGACCA | GAAGCACAGCCGTGAAGTTG |
| *Aalb*Opsin9 | TGACAGCAAGCAAACAGTCG | CATTGCCTTTGGTCCACATCG |
| *Aalb*Opsin10 | CACCTGATGGTGACCTACCG | GAATCGCTCCGGAACTCCC |
| *Aalb*Opsin12 | TGTGATCAGTAGCGGGAAGTC | GCAAAATGACCCACCCTTG |
| *Aaeg*Opsin1 | CGGCTTTGTCTTCGAGTCTG | ACAAATGCCGCCATTCTATTTC |
| *Aedes albopictus Actin* | CGGAAGAGCACCCAGTTCTC | TGTGTCATCTTCTCGCGGTTAG |
| *Aedes aegypti Actin* | GAACACCCAGTCCTGCTGACA | TGCGTCATCTTCTCACGGTTAG |
